# Repressive LTR Nucleosome Positioning by the BAF Complex Is Required for HIV Latency

**DOI:** 10.1371/journal.pbio.1001206

**Published:** 2011-11-29

**Authors:** Haleh Rafati, Maribel Parra, Shweta Hakre, Yuri Moshkin, Eric Verdin, Tokameh Mahmoudi

**Affiliations:** 1 Department of Biochemistry, Erasmus University Medical Center, Rotterdam, The Netherlands; 2 Gladstone Institute of Virology and Immunology, University of California–San Francisco, San Francisco, California, United States of America; Fred Hutchinson Cancer Research Center, United States of America

## Abstract

Persistence of a reservoir of latently infected memory T cells provides a barrier to HIV eradication in treated patients. Several reports have implicated the involvement of SWI/SNF chromatin remodeling complexes in restricting early steps in HIV infection, in coupling the processes of integration and remodeling, and in promoter/LTR transcription activation and repression. However, the mechanism behind the seemingly contradictory involvement of SWI/SNF in the HIV life cycle remains unclear. Here we addressed the role of SWI/SNF in regulation of the latent HIV LTR before and after transcriptional activation. We determined the predicted nucleosome affinity of the LTR sequence and found a striking reverse correlation when compared to the strictly positioned in vivo LTR nucleosomal structure; sequences encompassing the DNase hypersensitive regions displayed the highest nucleosome affinity, while the strictly positioned nucleosomes displayed lower affinity for nucleosome formation. To examine the mechanism behind this reverse correlation, we used a combinatorial approach to determine DNA accessibility, histone occupancy, and the unique recruitment and requirement of BAF and PBAF, two functionally distinct subclasses of SWI/SNF at the LTR of HIV-infected cells before and after activation. We find that establishment and maintenance of HIV latency requires BAF, which removes a preferred nucleosome from DHS1 to position the repressive nucleosome-1 over energetically sub-optimal sequences. Depletion of BAF resulted in de-repression of HIV latency concomitant with a dramatic alteration in the LTR nucleosome profile as determined by high resolution MNase nucleosomal mapping. Upon activation, BAF was lost from the HIV promoter, while PBAF was selectively recruited by acetylated Tat to facilitate LTR transcription. Thus BAF and PBAF, recruited during different stages of the HIV life cycle, display opposing function on the HIV promoter. Our data point to the ATP-dependent BRG1 component of BAF as a putative therapeutic target to deplete the latent reservoir in patients.

## Abstract 2

The SWI/SNF BAF chromatin remodeling complex generates a repressive nucleosome structure at the HIV LTR conducive to establishment and maintenance of HIV latency, while PBAF augments HIV transcription.

## Author Summary

Despite the effectiveness of antiretroviral medication, the HIV virus persists in resting memory T cells of infected patients in a latent state, providing the main impediment to eradication of the virus. In this article, we examined the molecular mechanism responsible for the establishment and maintenance of HIV latency and its re-activation, and uncovered the role played in this process by the SWI/SNF class of chromatin remodeling complexes, which use energy from ATP to alter the structure of chromatin. We show that two distinct sub-classes of SWI/SNF, BAF and PBAF, play functionally opposing roles in distinct steps of the HIV promoter (or long terminal repeat, LTR) transcription cycle. The PBAF complex augments transcription of the LTR by the viral transactivator Tat. In contrast, the distinct BAF complex generates a chromatin structure at the LTR that is energetically unfavorable with respect to the intrinsic histone-DNA sequence preferences. Specifically, we find that BAF positions a repressive nucleosome immediately downstream of the HIV transcription start site, abrogating transcription, and in this way contributes to the establishment and maintenance of HIV latency. Our data describe a novel molecular mechanism for the establishment and maintenance of HIV latency, and we identify the catalytic subunit of BAF, the enzyme BRG1, as a putative molecular target to deplete the latent reservoir in infected patients.

## Introduction

After host cell infection and entry into the nucleus, the Human immunodeficiency virus (HIV-1) DNA integrates into the host genome as a chromatin template. Through unclear mechanisms, a very small percentage of infected T cells become latent. Despite the successes of modern Highly Active Anti-Retroviral Therapy (HAART) in suppressing viral replication, the presence of latently infected resting memory CD4+ T cells provides the main impediment to curing HIV [1–3]. Infected patients must receive continuous HAART, as treatment interruption results in rapid rebound of viremia [4]. Latent HIV-1 infected resting memory CD4+ T cells harbor replication competent virus, which is blocked at the level of transcription.

Transcription of the HIV-1 virus is driven by the LTR and is restricted in vivo. Regardless of the position of virus integration in the host genome, within the 5′LTR, the nucleosomes are strictly deposited at specific positions [5–7]. Chromatin organization of the HIV-1 provirus characterized by nuclease digestion of intact nuclei of infected cells under basal conditions demonstrates the presence of at least three precisely positioned nucleosomes, nuc-0, nuc-1, and nuc-2 and their intervening nucleosome-free regions [5,6]. In particular, nuc-1, the nucleosome positioned immediately downstream of the transcription start site, is repressive to transcription and is surrounded by two large domains of nucleosome-free DNA. Following activation, nuc-1 becomes rapidly and specifically disrupted [5,8].

To overcome nucleosome mediated repression, the cell uses at least two mechanisms to increase the accessibility of DNA sequences embedded within nucleosomes. The first is through the action of enzymatic complexes which covalently modify histones. Histone modifying complexes are thought to regulate transcription at the HIV LTR. For example, HDAC1 is recruited to and represses transcription at the LTR [9–11]. Following activation, histone acetylation surrounding nuc-1 has been demonstrated to increase significantly, concomitant with removal of HDAC [7,10,12,13]. Many histone-modifying enzymes have been shown to be recruited to the LTR by the HIV transactivator Tat and/or by host cell transcription factors, whose consensus binding sites are present on the LTR. Tat itself is subject to distinct modifications by various factors (including p300/CBP, PCAF, hGCN5, SIRT1, PRMT5, SETDB1, SETDB2, SET7/9 KMT7) [14,15], a mechanism to modulate its interaction with the many cofactors Tat recruits to the LTR.

**Fig 1:**
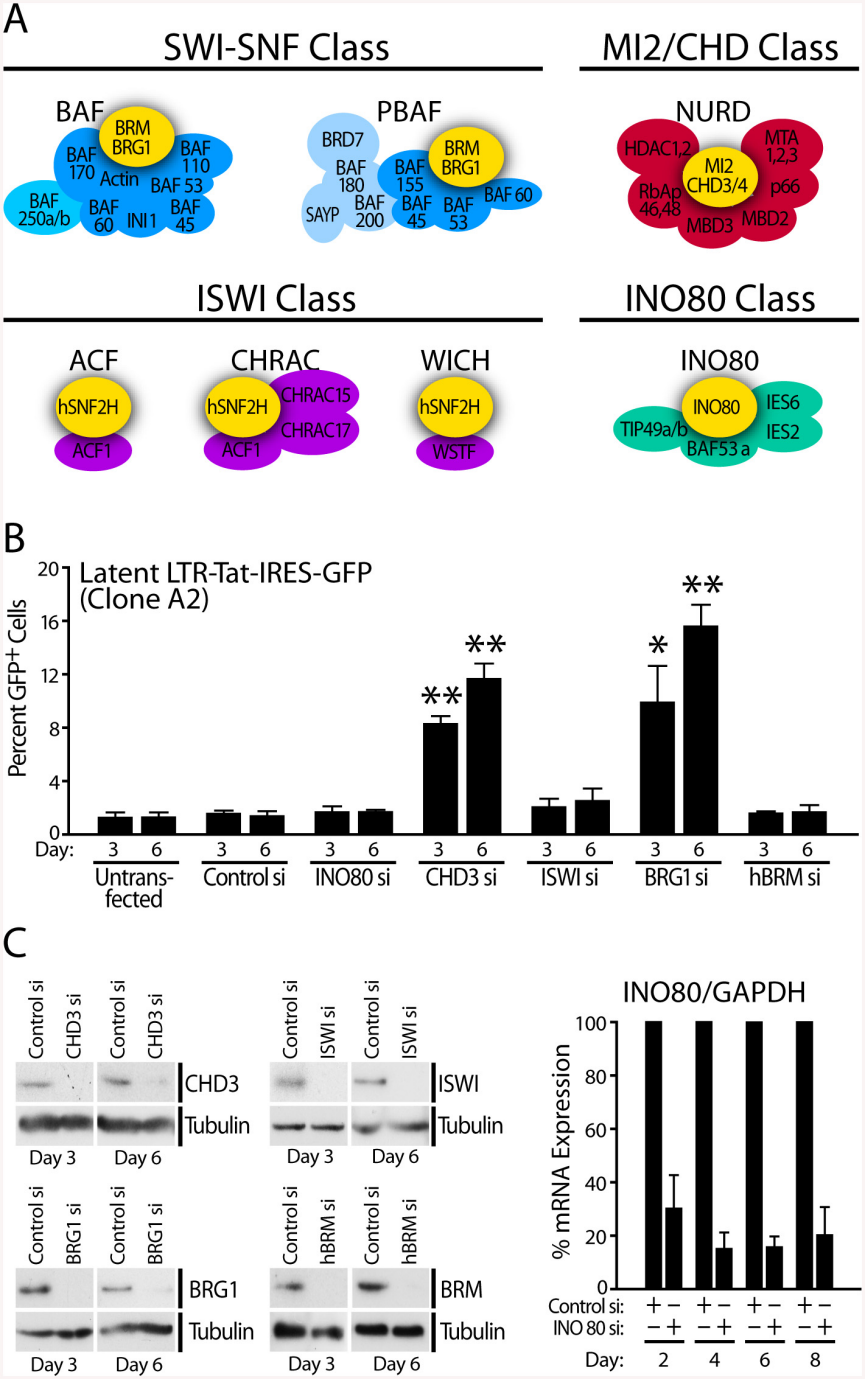
SWI/SNF and MI2 family of ATP-dependent chromatin remodeling enzymes are involved in LTR repression. (A) Four distinct classes of mammalian ATP-dependent chromatin remodeling complexes distinguished by their catalytic subunits, BRG-1/BRM (SWI/SNF), ISWI, INO80, or CHD3/4 (MI2). (B) RNAi depletion of ATPase subunit of each class of ATP-dependent chromatin remodeling complexes as indicated in J-Lat A2 cells containing an integrated latent LTR-Tat-IRES-GFP virus. Cells were nucleofected with control siRNA or siRNAs targeting INO80, CHD3, ISWI, BRG1, and BRM as indicated. GFP expression was monitored by FACS at indicated times post-transfection and is presented as % GFP-positive cells. (C) Western blot analysis to demonstrate depletion of individual ATPase subunits as indicated 3 and 6 d after transfection of siRNAs. RT-PCR analysis indicated stable depletion of INO80 mRNA up to 8 d after siRNA transfection. Error bars represent the SEM of three independent experiments. * *p* < 0.05, ** *p* < 0.01.

The second mechanism for altering DNA accessibility within repressive nucleosomes is via enzymatic complexes, which use energy from ATP hydrolysis to alter the structure of chromatin [16,17]. One family of remodeling complexes, SWI/SNF, contains either Brahma (BRM) or the closely related BRG1 as its catalytic subunit and shares most common subunits [16–22]. At least two biochemically distinct SWI/SNF complexes with different functions have been described and are called BAF and PBAF. The PBAF complex contains either BRG1 or BRM together with the PBAF-specific subunits BAF180, BAF200, SAYP, and Brd7, but lacks BAF250 [23–27]. The BAF complex contains either BRG1 or BRM together with the BAF-specific subunit BAF250, but lacks PBAF-specific subunits (Fig 1A) [28,29]. The presence of distinct isoforms of subunits such as BAF60 also increases the number of possible complexes [16,17]. BAF- or PBAF-specific subunits have been implicated in transcriptional activation by selective nuclear hormone receptors [24,28,30,31], in distinct roles in cell cycle control and mitosis [29,32], and the expression of interferon-responsive genes [26]. OSA, the Drosophila BAF-specific subunit, is required for repression of *Wingless* target genes [33]. BAF and PBAF subunit-specific polytene staining of Drosophila salivary glands indicates that the complexes are recruited to distinct targets [34]. These and other studies suggest that BAF and PBAF complexes perform distinct functions in transcription regulation.

In the immediate-early phase of HIV infection, cellular transcription factors activate transcription from the viral promoter in the 5′LTR, leading to accumulation of viral Tat protein, a potent transactivator. Tat binds TAR, an RNA stem-loop in the nascent viral RNA, and recruits a positive transcription elongation factor complex (pTEFb) containing CDK9 and cyclinT1. This recruitment leads to the phosphorylation of the carboxyl-terminal domain of RNA PolII and increased transcriptional elongation. In turn, more efficient transcription of the HIV genome, including Tat, generates a Tat-dependent positive feedback loop [15].

Tat also leads to the remodeling of nuc-1 [5,35], the nucleosome positioned immediately downstream of the transcription start site. We and others have reported that Tat recruits the SWI/SNF chromatin-remodeling complex to the HIV LTR to activate transcription [36–39]. SWI/SNF was also shown to promote HIV transcription elongation via a Tat-independent mechanism [40]. INI-1 (hSNF5), a core subunit to all SWI/SNF complexes, was first identified because of its interaction with HIV IN [41]. In addition to its direct involvement in Tat-mediated LTR activation [38,39], INI-1 was shown to restrict early steps of HIV infection [42] and to repress basal LTR activity [43]. Recently, the interaction between the HIV IN and SWI/SNF was suggested to functionally couple the processes of integration and remodeling necessary for integration into stable nucleosomes [44]. However, despite these studies on regulation of the HIV life cycle by SWI/SNF, the mechanism behind the seemingly contradictory involvement of SWI/SNF in regulating various stages of the HIV life cycle (i.e. integration, transcription activation, as well as repression) is not understood. Here we examined the mechanistic role played by SWI/SNF in the establishment and maintenance of HIV latency and its re-activation.

## Results

### The SWI/SNF and MI2 Family of ATP-Dependent Chromatin Remodeling Enzymes Are Involved in LTR Repression

We and other laboratories previously reported the requirement of SWI/SNF chromatin remodeling complexes on activation of HIV-1 LTR [36–40]. To investigate further the role of ATP-dependent remodelers on LTR regulation, we examined the effect of cellular ATP depletion by sodium azide (NaN3) on LTR activity and chromatin remodeling. We treated a Jurkat cell line containing an integrated LTR-GFP virus (S1B and S1C Fig) [45] as well as J-Lat A2 [46], containing an integrated latent LTR-Tat-IRES-GFP virus (S1D and S1E Fig) with increasing concentrations of NaN_3_. Surprisingly, in the absence of Tat expression, addition of NaN_3_ was associated with derepression of basal HIV promoter activity (S1C and S1E Fig). To determine whether this derepression was associated with chromatin remodeling of the HIV promoter, we used a restriction enzyme accessibility assay coupled to indirect end-labeling, as described [5]. In this assay, nuc-1 remodeling leads to increased accessibility of the restriction enzyme AflII to its recognition site, generating a novel restriction fragment (S1A Fig). Nuc-1 remodeling was assayed in untreated cells, in cells treated with PMA as a positive control, and in cells treated with NaN_3_ (S1B and S1D Fig). We observed nuc-1 remodeling in response to NaN_3_ at the same concentrations that induced HIV promoter derepression (S1C and S1E Fig). Thus, with the caveat that toxicity resulting from cellular ATP depletion by NaN_3_ may be accompanied by non-specific effects, these observations suggested that ATP dependent chromatin remodeling activity is required to suppress basal promoter activity.

To determine which family of ATP-dependent chromatin remodeling enzymes may be involved in LTR repression, we used siRNAs to deplete the expression of the catalytic subunit of each class of mammalian remodelers. There are four major families of remodelers, each named after their central ATPase: SWI/SNF (BRG1 or BRM), ISWI, CHD/MI2, and INO80 (Fig 1A) [16,17]. Specific siRNAs directed against each catalytic subunit were transfected via nucleofection into J-Lat A2 cells (Fig 1B) leading to the efficient depletion of each factor as shown by Western blot analysis for CHD3, ISWI, BRG1, and BRM and by RT-PCR for INO80 (Fig 1C). Depletion of CHD3 resulted in derepression of latent HIV LTR activity as measured by an increase in GFP expression (Fig 1B). In support of this observation, the CHD3 containing NuRD complex as well as the methyl CpG binding protein MBD2, which is another component of the NuRD complex, were shown to be involved in LTR repression [47,48]. The related CHD1 protein was also shown to repress the HIV LTR [49]. siRNA nucleofection had no non-specific effect on LTR-driven GFP expression. While depletion of INO80, ISWI, or BRM had no affect on LTR activity, we found robust de-repression of latent LTR upon BRG1 depletion. This suggested that a BRG1 containing SWI/SNF complex represses basal HIV promoter activity and may be required for maintaining latency.

### Repression of Basal HIV Promoter Activity by the BAF Complex

The HIV transactivator Tat recruits the SWI/SNF ATP-dependent chromatin-remodeling complex to the LTR to activate transcription [36–39]. The observation that the HIV preintegration complex interacts with the core SWI/SNF subunit INI-1/hSNF5 in the cytoplasm before integration [41,44,50] suggested that the SWI/SNF complex might also regulate the activity of the HIV LTR immediately after integration, before Tat accumulation. In support of this notion, INI-1 has been implicated in both LTR activation and repression [38,39,43].

**Fig 2:**
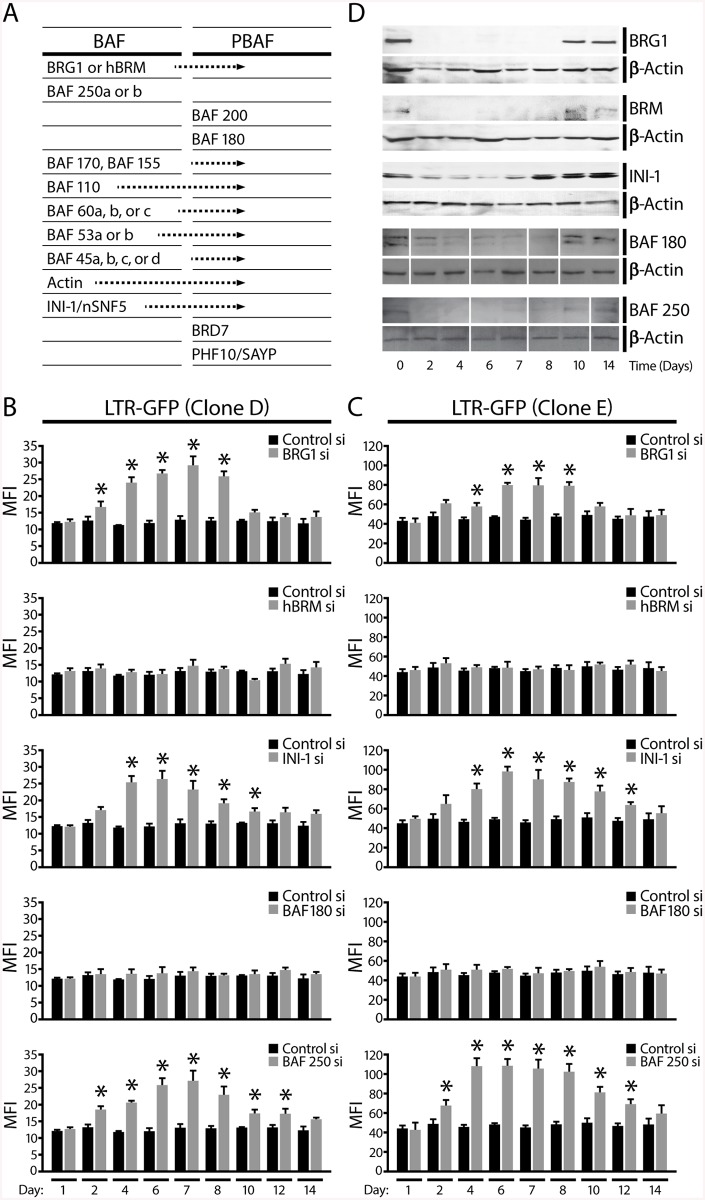
The BAF complex represses basal transcription at the HIV promoter. (A) Table of subunit composition of the two distinct SWI/SNF complexes, BAF and PBAF, in mammals. (B) GFP expression was monitored by flow cytometry at indicated times after siRNA transfection to measure HIV promoter activity. Results are presented as MFI for cells treated with a control siRNA or siRNAs specific for SWI/SNF subunits. (C) Same experiments as shown in (B) for clone D, with clone E, another Jurkat cell line containing an integrated LTR-GFP virus. Error bars represent the SEM of five independent experiments. * *p*<0.05. (D) Jurkat cells containing an integrated LTR-GFP virus (clone D) were transfected with control siRNA or siRNAs targeting various SWI/SNF complex subunits as indicated. Western blot analysis shows expression of each SWI/SNF subunit after its specific depletion 0, 2, 4, 6, 7, 8, 10, and 14 d after siRNA transfection with each specific antibody and β-actin loading control as indicated.

To examine this possibility, and determine which of the compositionally distinct SWI/SNF complexes, BAF or PBAF (Fig 2A), may be responsible for repressing HIV LTR, we used siRNAs to deplete the expression of selected subunits, in two clonal Jurkat cell lines (clones D and E) that contain single integrations of the minimal HIV genome, LTR-GFP virus lacking Tat, and expressing low levels of GFP (Fig 2B and 2C). To dissect the requirements of distinct BAF/PBAF complexes in basal LTR-driven, Tat-independent transcription, siRNAs directed against the core subunits BRG1, BRM, INI-1, the PBAF-specific subunit BAF180, and the BAF-specific subunit BAF250a were transfected via nucleofection leading to the efficient depletion of each factor as shown for Jurkat clone D by Western blot analysis (Fig 2D). We used BAF180 depletion to examine the effect of PBAF on LTR-driven transcription as depletion of the PBAF-specific BAF200 was previously reported to result in disruption of the complex [26]. To ensure that the stability of the complex is not compromised when one component of the complex is depleted by siRNA, we examined the protein levels of the other SWI/SNF subunit by Western blot analysis after depletion of each subunit. SWI/SNF subunit protein levels were unaffected by siRNA depletion of specific subunits (S2 Fig). HIV promoter activity was monitored using flow cytometry by measuring changes in Mean Fluorescent Intensity (MFI) of the low basal GFP fluorescence in Jurkat clones D and E for 2 wk after siRNA depletion of SWI/SNF subunits (Fig 2B and 2C). Depletion of BRG1, INI-1, and the BAF-specific BAF250a resulted in derepression of HIV promoter activity as measured by an increase in GFP expression monitored over 14 d. In contrast, depletion of BRM or the PBAF-specific BAF180 did not affect LTR activity (Figs 2B, 2C and 1B). Derepression of HIV LTR activity peaked at approximately 8 d followed by de novo repression. LTR derepression was tightly correlated with depletion of SWI/SNF subunits as shown by Western blot analysis (Fig 2D). Derepression of basal HIV promoter activity in response to BAF250a depletion suggested that the BAF complex, which is defined by the unique BAF250 subunit, is required for repression of basal LTR activity. In agreement with this model, depletion of BAF180, unique to PBAF, had no effect on basal repressed HIV promoter activity. Identical results observed with both Jurkat clones containing integrated HIV LTR-GFP demonstrated that this effect occurs independent of the two distinct integration sites.

### Latent HIV Is Derepressed Upon BAF Depletion

**Fig 3:**
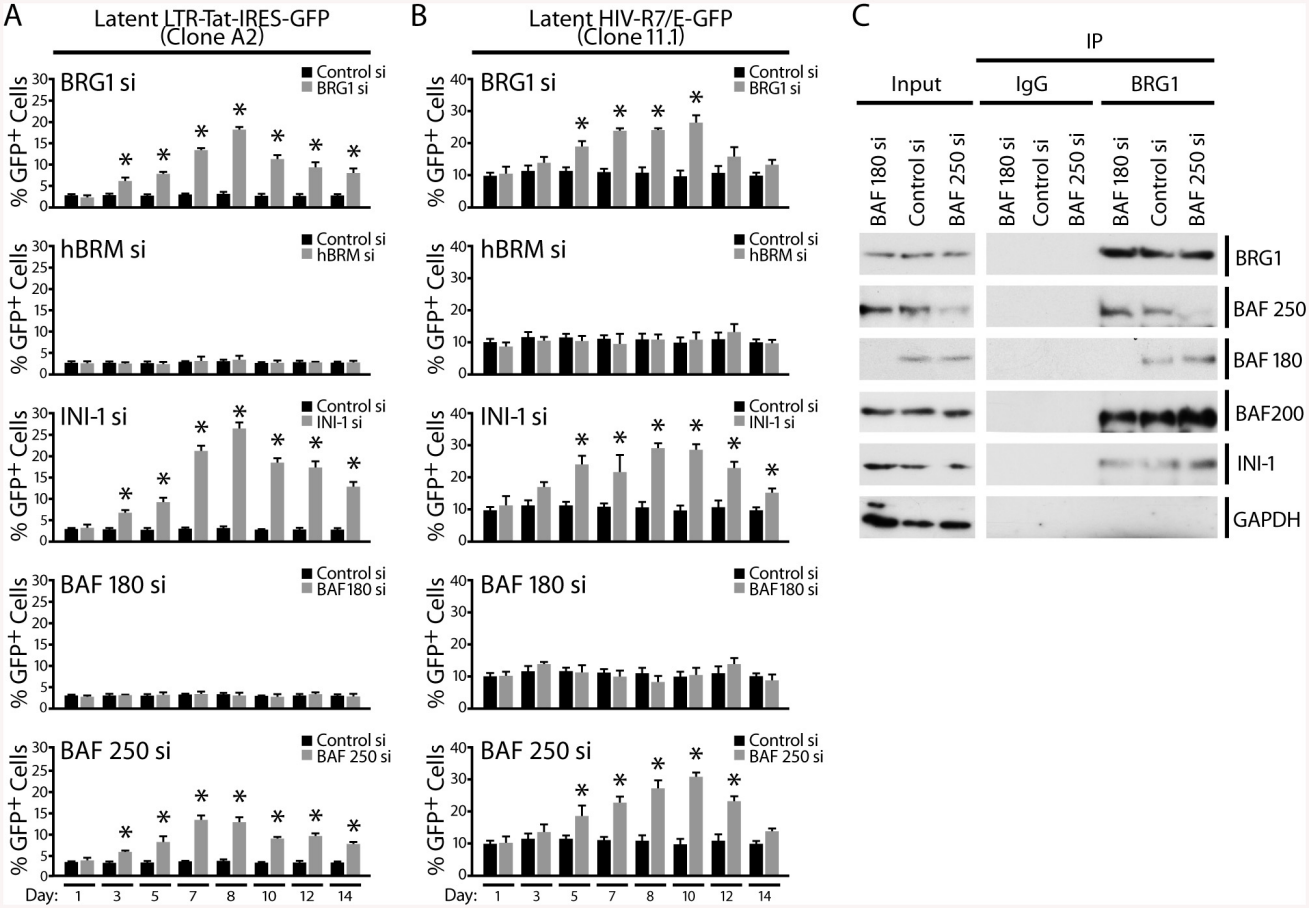
Reactivation of latent HIV mediated by knockdown of BAF subunits. (A) J-Lat A2 cells latently infected with LTR-Tat-IRES GFP virus were transfected with either control siRNA or siRNAs targeting various SWI/SNF subunits as indicated. GFP expression was monitored by FACS at the times indicated after transfection and is expressed as % GFP-positive cells. (B) J-Lat 11.1, clone of Jurkat cells containing a latent integrated full-length HIV virus harboring GFP in place of Nef, was transfected with either control siRNA or siRNAs targeting various SWI/SNF subunits as indicated. GFP expression was monitored by FACS at the times indicated after transfection and is expressed as % GFP-positive cells. Error bars represent the SEM of five independent experiments. * *p* < 0.05. (C) The BRG-1 complex remains intact following depletion of BAF or PBAF-specific subunits. BRG-1 was immunoprecipitated from J-Lat A2 cells transfected with control siRNA or siRNA targeting BAF250 or BAF180, and its associated proteins were examined by SDS-PAGE and Western blotting with antibodies against BRG-1 itself, the BAF or PBAF-specific subunits BAF250a, BAF180 and BAF200, the core subunit INI-1 and GAPDH as control.

Suppression by the BAF complex was also observed during a latent HIV infection. SWI/SNF subunits were depleted as described above by siRNA in two Jurkat clonal cell lines, J-Lat A2 and J-Lat 11.1, which harbor latent HIV [46]. J-Lat A2 contains an integrated latent LTR-Tat-IRES-GFP virus (Fig 3A), and J-Lat 11.1 contains a full-length HIV-1 genome expressing GFP in place of Nef (Fig 3B). J-Lat cells are not transcribing, and are therefore GFP negative in the latent state. Because of the absence of Tat expression, this system is ideal to examine the role of chromatin modulators in derepression of latent LTR, which leads to an increase in the percentage of LTR-driven GFP expressing cells. We depleted core and BAF/PBAF-specific SWI/SNF subunits from J-Lat cells by siRNA transfection. To ensure that the BRG-1 complex remains intact following depletion of BAF- or PBAF-specific subunits, we immunoprecipitated BRG-1 from J-Lat A2 cell lysates, which were either undepleted or depleted of BAF250 or BAF180 and probed for the presence of BAF/PBAF subunits by Western blot analysis (Fig 3C). Depletion of BAF180 or BAF250 did not affect the binding of the other core or specific SWI/SNF subunits to the BRG-1 immunoprecipitated complexes, supporting the notion that there is no cross-talk between these distinct SWI/SNF complexes. In both J-Lat cell lines, depletion of BRG1, INI-1, or the BAF-specific subunit BAF250a resulted in derepression of HIV expression, as demonstrated by an increase in percent GFP-positive cells. GFP expression peaked 6–10 d after siRNA nucleofection (Fig 3A and 3B) and inversely correlated with expression of BRG1, INI-1, and BAF250a (unpublished data). To address the perceived lag between factor depletion and GFP detection by FACS analysis, which peaked at 6–10 d following siRNA transfection, we performed RT-qPCR time-course analysis of GFP mRNA expression in J-Lat A2 cells after siRNA depletion of BAF250 and BRG-1 (S3A Fig). GFP mRNA was significantly induced as early as 2 d post-siRNA transfection, at the same time point in which significant protein depletion is achieved, and peaked at 4–5 d following siRNA transfection. Thus the delay in GFP detection by FACS appears to be a matter of accumulation of GFP protein over background levels. We also examined the effect of depletion of BAF250b, a BAF-specific complex component also expressed in Jurkat cells, which is functionally and biochemically distinct from the BAF250a complex [51], on LTR regulation in J-Lat A2 cells (S3B Fig). While depletion of BAF250a and BRG-1 caused robust expression of GFP mRNA, depletion of BAF250b had no effect on LTR transcription. These results demonstrated that the BAF250a-containing BAF complex is specifically required for maintenance of repression of latent HIV.

### K50,51 Acetylated Tat Coimmunoprecipitates with and Requires PBAF for Transactivation of HIV Promoter

**Fig 4:**
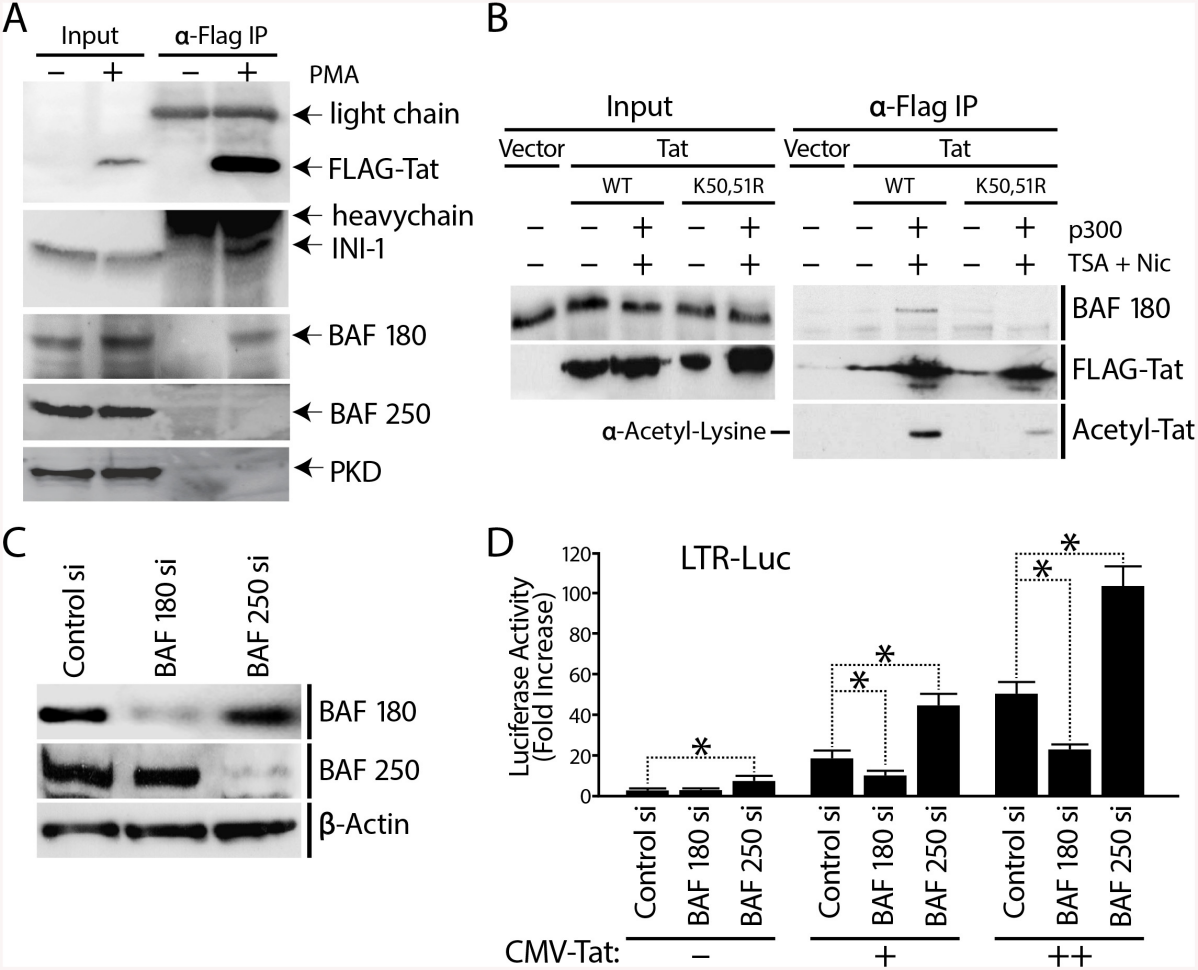
PBAF, recruited by K50K51 acetylated Tat, is a co-factor for Tat activation of the HIV promoter. (A) J-Lat A2 cells containing an integrated LTR-Tat-FLAG-GFP were stimulated with PMA to induce expression of Tat-FLAG. Tat was immunoprecipitated from untreated or PMA-stimulated cell lysates and its associated proteins were examined by SDS-PAGE and Western blotting with antibodies against the BAF- or PBAF-specific subunits BAF250a and BAF180, and protein kinase D-1 and 14-3-3 as controls. (B) Tat co-immunoprecipitation with BAF180 is modulated by Tat acetylation. Tat (wild-type or K50R/L51R) was immunoprecipitated using anti-FLAG antibody and analyzed by Western blotting using antibody specific for BAF180. Tat acetylation levels were assessed using an anti-acetyl lysine antibody. All proteins were expressed at similar levels under the different experimental conditions as shown by the Inputs. (C) 1G5 Jurkat cells containing integrated LTR-Luciferase (LTR-Luc) were nucleofected with siRNAs against BAF180, BAF250, or with a control siRNA pool. Expression of BAF180, BAF250, and β-actin was analyzed by Western blotting after depletion of either BAF180 or BAF250. (D) Transactivation of the HIV promoter by Tat is reduced in the absence of BAF180. 48 h after siRNA depletion of BAF180 or BAF250, cells were re-transfected with either a control or Tat-expression vector (CMV-driven), and luciferase assay performed after 24 h. Error bars represent the SEM of three independent experiments. * *p*<0.05. (D).

We and others reported that a SWI/SNF complex associates with Tat to activate the HIV promoter [36–39]. A recent study found that depletion of neither the PBAF-specific BAF180 nor BAF250a had any effect on Tat-mediated LTR activation, while another PBAF-specific component BAF200 was required for Tat activation of the LTR [52]. We and other laboratories have found synergism between the acetyltransferase p300 and SWI/SNF in LTR activation. This activation was found to be dependent on Tat acetylation [36,38]. Our novel data indicating derepression of LTR transcription in response to depletion of BAF-specific subunits suggested that the BAF complex is recruited to the HIV promoter independently of Tat. We therefore sought to determine which SWI/SNF complex, PBAF or BAF, is specifically recruited and required by Tat to activate transcription, and whether Tat acetylation is necessary for this interaction. We performed immunoprecipitation experiments in the J-Lat A2 cells, which express no Tat under basal conditions and epitope-tagged Tat (Tat-FLAG) after reactivation of HIV by PMA (Fig 4A). Using this system we have shown previously that Tat coimmunoprecipitated with INI-1, BRG1, and β-actin, three core subunits shared between BAF and PBAF complexes [38]. Importantly, Tat co-immunoprecipitated with BAF180, but not with BAF250a or the unrelated protein kinase D (Fig 4A). Thus, Tat interacts specifically with the BAF180-containing PBAF complex.

To determine whether the interaction of Tat with PBAF is modulated by acetylation, we cotransfected 293T cells with wild-type or K50R/K51R mutant Tat in the presence or absence of a p300 expression vector (Fig 4B). To prevent Tat deacetylation, cells were treated with nicotinamide and trichostatin A, inhibitors of class III and class I and II histone deacetylases, respectively. We found that Tat association with BAF180 increased in the presence of p300 as shown by co-immunoprecipitation of BAF180 with Tat (Fig 4B). The same treatment markedly increased Tat acetylation (Fig 4B). Importantly, the Tat(K50R/K51R) mutant did not display increased affinity for BAF180 in response to p300 (Fig 4B). Mutation of Tat residues Lys^50^ and Lys^51^ to arginine decreased Tat acetylation significantly, but not completely, consistent with the existence of other Tat acetylation sites [53]. These results support a model in which p300 acetylated Tat specifically recruits the PBAF complex to the HIV LTR.

Our data suggested that the distinct SWI/SNF complexes BAF and PBAF might play temporally distinct roles in HIV transcription: Tat-independent basal repression of promoter activity by BAF and Tat-dependent activation of promoter activity by PBAF. To test this model, we examined the effect of selective knockdown of BAF250a and BAF180, unique to each complex, on Tat-independent and Tat-dependent HIV transcription. Jurkat 1G5 cells contain an integrated LTR-luciferase reporter construct and allow convenient monitoring of HIV promoter activity [54]. Cells were first transfected with siRNAs against either BAF180 or BAF250a leading to their efficient depletion (Fig 4C). To probe the effect of BAF/PBAF depletion on Tat-dependent LTR transcription, we introduced Tat exogenously by re-transfecting the cells with an expression vector for Tat or an empty control vector. Nucleofection of siRNA or control vectors had no non-specific effect on LTR-driven luciferase expression (S4B Fig). As seen before (Figs 2 and 3), depletion of BAF250a and not BAF180 resulted in an increase in basal promoter activity, confirming the repressive role of BAF250a in Tat-independent LTR-driven, luciferase expression in 1G5 cells (Fig 4D). In the presence of Tat, depletion of BAF250a resulted in a significant increase in Tat-mediated activation, suggesting a synergistic effect between Tat expression and loss of the repressive BAF complex on LTR activity. In contrast, depletion of BAF180 suppressed Tat-dependent HIV promoter activity at both concentrations of Tat tested (Fig 4D). CMV-driven Tat expression, driven by the CMV promoter, was not affected by depletion of BAF180 or BAF250a (S4A Fig). We also compared the effect of both wild type and K50,51R mutant Tat in 1G5 cells containing or depleted of BAF180 or BAF250a (S4B Fig). While depletion of BAF180 suppressed optimal LTR activation by wild type Tat, it had no significant suppressive effect on LTR activation by K50,51R mutant Tat, supporting the notion that acetylated Tat recruits PBAF to facilitate LTR activation. Altogether, these results indicate that PBAF is specifically required for optimal Tat-mediated transactivation of the HIV promoter.

### Direct Binding of Distinct SWI/SNF Complexes to the HIV Promoter Before and After Transcriptional Activation

**Fig 5:**
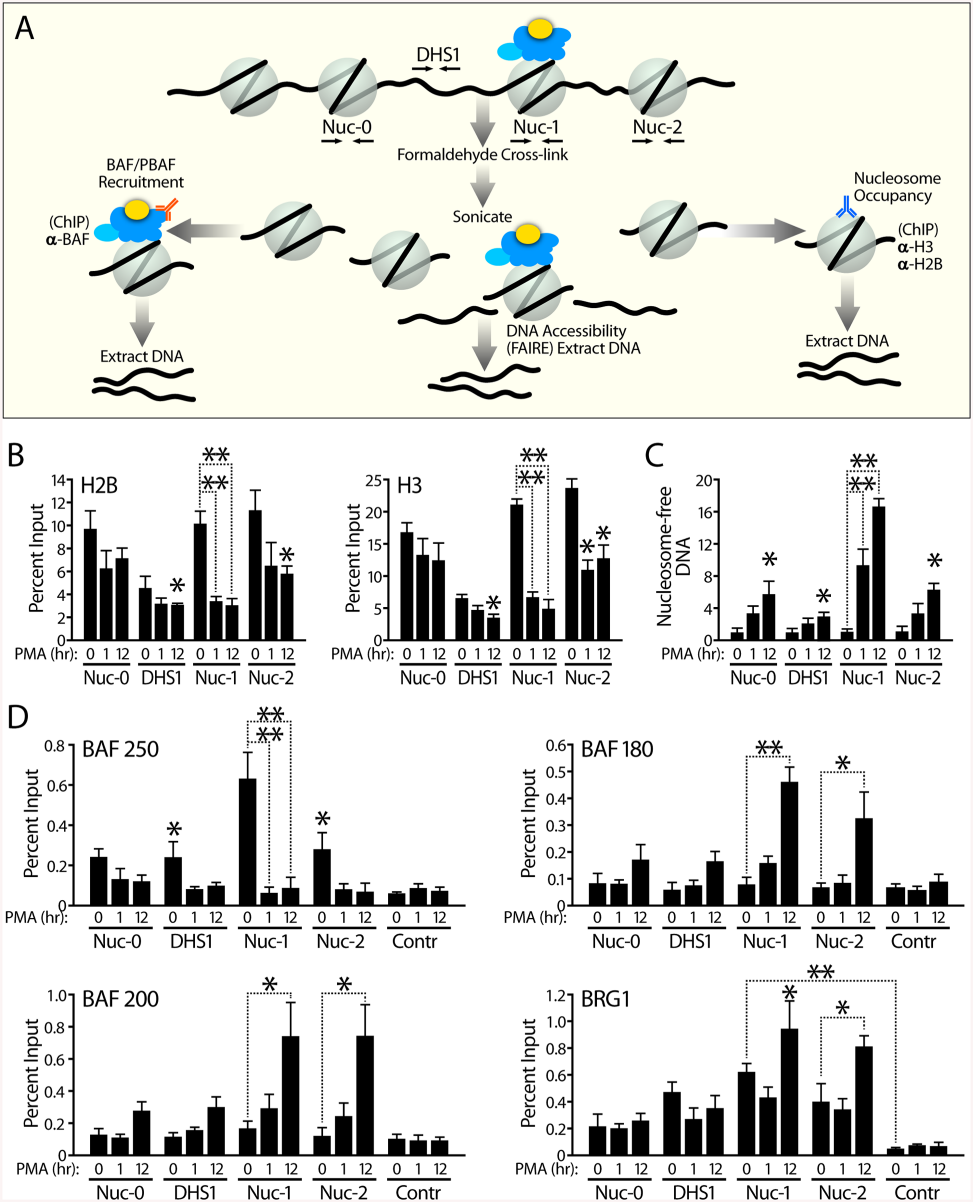
Direct binding of distinct SWI/SNF complexes to the HIV promoter before and after transcriptional activation. (A) Schematic representation of strategy to explore nucleosome position changes and enrichment of SWI/SNF at the HIV LTR in its repressed state and after PMA stimulation. J-Lat 11.1 cells at 0, 1, and 12 h after PMA addition were crosslinked and sonicated to yield fragments of approximately 150 bp. DNA accessibility was monitored by FAIRE while nucleosome occupancy was determined by histone H3 and H2B ChIPs. To determine enrichment of SWI/SNF complexes, we performed ChIPs with antibodies specific for BAF250, BAF180, BAF200, and BRG1. (B) PMA stimulation causes a reduction in histone density over HIV nuc-1 as determined by H3 and H2B ChIPs. Histone ChIPs are presented as percent immunoprecipitated DNA over input. (C) PMA stimulation is accompanied by an increase in DNA accessibility over the positioned nuc-1 of the HIV LTR. FAIRE results are presented as fold change respective to the unstimulated value (normalized to 1) for each primer pair. (D) BAF250a-specific BAF complex directly binds to nuc-1 in its repressed state, while BAF180 and BAF200-specific PBAF is recruited to nuc-1 upon PMA stimulation. SWI/SNF subunit ChIPs are presented as percent immunoprecipitated DNA over input. Immunoprecipitated DNA from ChIPs and phenol:chloroform extracted DNA from FAIRE were analyzed by qPCR using primer pairs specific for the HIV LTR nuc-0, DHS1, nuc-1, and nuc-2 regions, and control region amplifying upstream of the Axin2 gene. For all ChIP and FAIRE experiments, error bars represent the SEM of at least three independent experiments. * *p* < 0.05, ** *p* < 0.01. We depict results for J-Lat 11.1. Similar results were obtained for J-Lat A2 (S5 Fig).

Our results suggested that distinct subunits of the SWI/SNF complexes are recruited to the HIV promoter in the absence and presence of Tat. We used a combinatorial approach based on formaldehyde crosslinking to determine the nucleosome density and DNA accessibility at the LTR, and to demonstrate direct interaction of SWI/SNF subunits with the HIV promoter (Fig 5A). Chromatin from J-Lat 11.1, containing a latent integrated full-length HIV virus (Fig 5) [46] or J-Lat A2 (S5 Fig), was prepared from cells at 0, 1, and 12 h after addition of PMA to culture supernatants. Following formaldehyde crosslinking, we fragmented chromatin by sonication, because it allows the isolation of both nucleosome-bound DNA and nucleosome-free DNA. To be able to distinguish between the nuc-0, DHS1, nuc-1, and nuc-2 regions within the LTR by qPCR, we sonicated the chromatin extensively to obtain small fragments of approximately 100–250 base pairs in size. To determine nucleosome occupancy, we performed chromatin immunoprecipitation experiments (ChIPs) using antibodies against histones H2B and H3 (Fig 5A and 5B). To independently map regions within the HIV LTR that are depleted of nucleosomes, we used FAIRE (Formaldehyde Assisted Isolation of Regulatory Elements) (Fig 5A and 5C). FAIRE relies on a phenol-chloroform extraction to isolate “nucleosome-free” DNA fragments that are not cross-linked to histones, and thus provides a complementary approach independent of antibodies, to examine chromatin structure. To examine recruitment of SWI/SNF complexes to the LTR, we subjected the sonicated chromatin to immunoprecipitation with BRG1, the BAF-specific BAF250a, and the PBAF-specific BAF200 and BAF180 antibodies (Fig 5A and 5D). The immunoprecipitated or phenol:chloroform extracted DNA was analyzed by qPCR with primer pairs specific for the nuc-0, DHS1, and nuc-1 regions of the HIV promoter (Fig 5A).

As shown in Fig 5C, PMA stimulation caused a dramatic increase in DNA accessibility, observed over the positioned repressive nuc-1 and encompassing nuc-0, DHS1, and nuc-2 albeit to a lesser extent. This observed increase in DNA accessibility in response to PMA stimulation is accompanied by a loss of histones bound to nuc-1 as determined by H2B and H3 ChIPs (Fig 5B). The ChIP results using antibodies directed against either histone H3 or H2B correlated well with each other. In agreement with the functional data discussed above and the proposed repressive role of BAF on the HIV promoter, we detected the BAF-specific subunit BAF250 and BRG1 bound to the HIV promoter nuc-1 under basal conditions. A remarkable switch in specific SWI/SNF subunits occurred in response to PMA: the BAF-specific subunit BAF250 was lost from the HIV promoter, while the PBAF-specific subunits, BAF180 and BAF200, were recruited during the transcriptional activation of the HIV promoter (Fig 5D). Compared to the control locus, BRG1 was enriched on the HIV promoter in its repressed state and slightly enriched in response to PMA stimulation. Thus, the PBAF complex is absent from the HIV promoter under basal conditions but recruited to the HIV promoter in response to PMA stimulation, while BAF is directly associated with the HIV LTR and required to maintain repression of the HIV promoter in the absence of Tat.

### BAF Is Essential for Positioning the Repressive nuc-1 of HIV LTR

**Fig 6:**
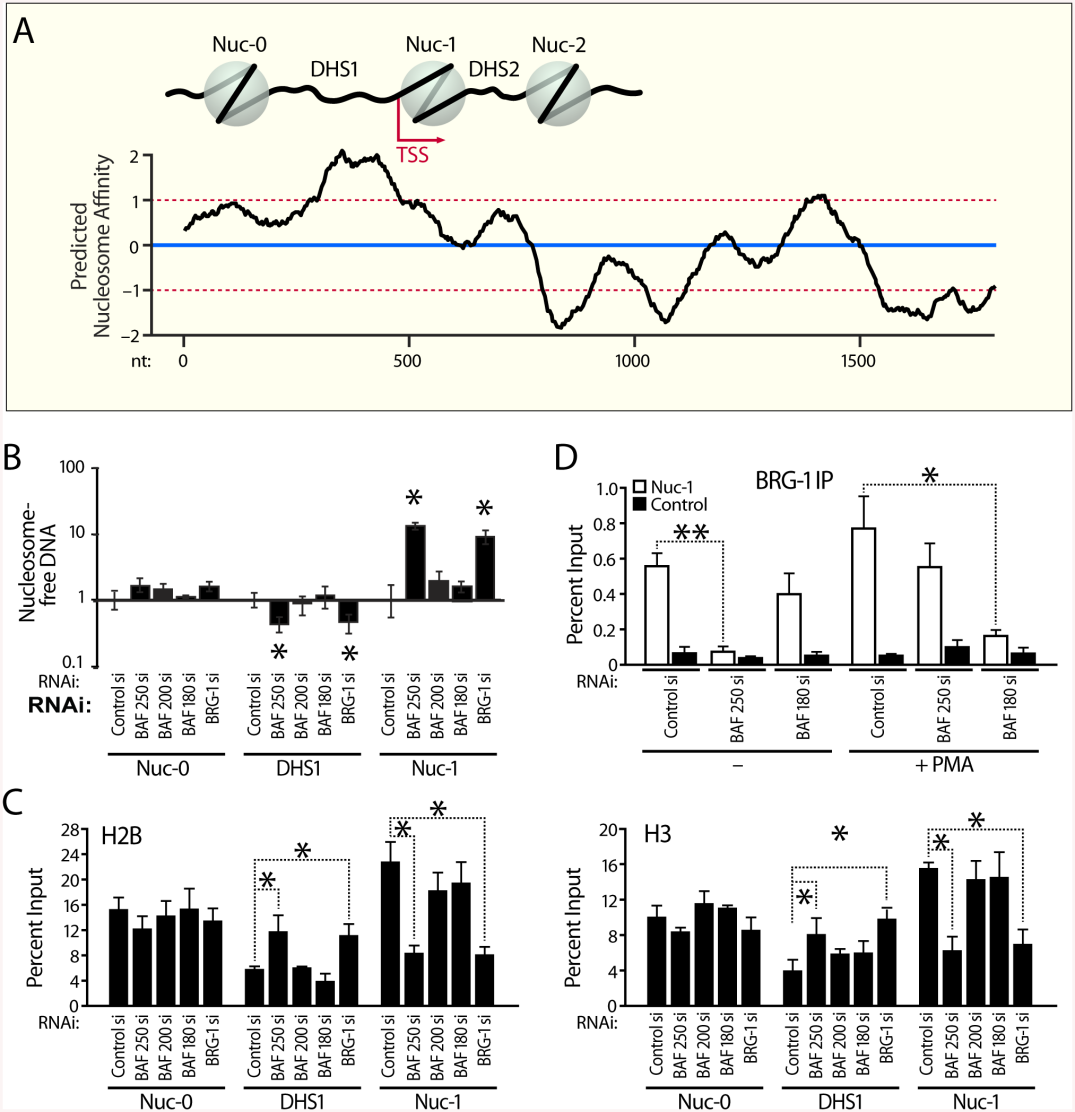
BAF represses HIV transcription by positioning nuc-1 of the HIV LTR. (A) Location of the strictly positioned nucleosomes correlates negatively with the predicted histone binding affinity score (nucleosome score) of the DNA sequence encompassing the HIV LTR. Predicted nucleosome affinity for HIV nucleotide sequence 1–1800 was determined using the algorithm described [55]. Similar results were obtained with an alternative algorithm described (S6 Fig) [56,57]. Means and standard deviations for nucleosome score at insertion sites are indicated by blue (mean) and red lines (mean ± SD) and give reference to known genomic sites of HIV integration [58]. (B) Depletion BAF250 and BRG1 results in a peak in DNA accessibility over positioned nuc-1 and decreased accessibility over DHS1 of the HIV LTR. J-Lat 11.1 cells were nucleofected with either nontargeting siRNA or siRNAs targeting individual SWI/SNF subunits as indicated and subjected to FAIRE. FAIRE results are presented as fold change respective to value obtained for control siRNA transfected cells (normalized to 1) for each primer pair. (C) Depletion of BAF results in a reduction in histone density over the positioned LTR nuc-1 as determined by H3 and H2B ChIPs. (D) BAF180 and BAF250 are distinctly required for targeting of the BRG1 complex to the activated and silenced LTR nuc-1, respectively. Depletion of BAF250 abrogates targeting of the repressive BRG-1 complex while BAF180 depletion interferes with recruitment of BRG1 to nuc-1 in response to PMA stimulation. J-Lat 11.1 cells were nucleofected with either nontargeting siRNA or siRNAs targeting BAF250 or BAF180 as indicated. Depleted cells were then subjected to ChIPs using an antibody specific for BRG1. BRG1 ChIPs were analyzed by qPCR using primer pairs specific for the LTR nuc-1 and control region amplifying upstream of the Axin2 gene and are presented as percent immunoprecipitated DNA over Input. For all ChIP and FAIRE experiments, error bars represent the SEM of at least three independent experiments. * *p* < 0.05, ** *p* < 0.01. We depict results for J-Lat 11.1. Similar results were obtained for J-Lat A2 (S6 Fig).

We next investigated the mechanism by which the BAF complex represses HIV LTR activity. One intriguing possibility was that nuc-1 positioning downstream of the transcription start site might be an active process driven in part by BAF activity, ATP hydrolysis. Our observation that nuc-1 becomes remodeled upon ATP depletion (S1 Fig) was consistent with this model. First we examined the propensity for nucleosome formation of the DNA sequence encompassing the first 1,800 base pairs of the HIV LTR including the positioned nucleosomes nuc-0, nuc-1, nuc-2, and the DNase hypersensitive sites (DHS1 and DHS2) in between (Fig 6A). We determined histone binding affinity score (nucleosome score) as the log likelihood ratio for the given region to be a nucleosome versus a linker using NuPoP software tool [55]. Similar results were obtained with an alternative algorithm (S6A Fig) [56,57]. The mean value for histone affinity score as shown by the blue line and standard deviation shown in red are indicated as reference to the known preferred genomic sites of HIV integration [58]. Comparison of the predicted affinities to the known in vivo positioning of nucleosomes within the HIV LTR [5,6] demonstrated a remarkable opposite correlation (Fig 6A). The region with the highest predicted propensity for nucleosome formation encompasses the DHS1, the nucleosome-free region found between the positioned nucleosomes nuc-0 and nuc-1. Conversely, sequences encompassing nuc0 and nuc-1 have a lower nucleosome score than the DHS1. The positioned nuc-2 and DHS2 separating it from nuc-1 also appear to be the mirror image of the nucleosome score predicted by their DNA sequences (Fig 6A). The negative correlation between predicted and actual nucleosome positioning in the HIV LTR together with the presence and functional requirement of BAF on nuc-1 in the repressed state suggested that BAF may counteract DNA sequence effects to place the repressive nuc-1 in a thermodynamically sub-optimal position.

We next sought to probe the role of BAF in nucleosome positioning within the LTR using a siRNA approach to selectively deplete BAF and PBAF subunits. To examine the impact of depletion of BAF on local chromatin structure at the HIV LTR, we first performed FAIRE experiments (Fig 6B and S6B Fig). Using FAIRE we assessed changes in DNA accessibility at the HIV LTR following depletion of BAF and PBAF subunits (Fig 6B). Strikingly, there was a sharp peak in DNA accessibility at nuc-1 following loss of BAF250 and BRG1, accompanied by a decrease in DNA accessibility over DHS1 (Fig 6B and S6B Fig). In contrast, knock-down of BAF180 or BAF200 had no significant effect on DNA accessibility at the HIV LTR (Fig 6B and S6B Fig). We next examined nucleosome density by ChIP-qPCR using antibodies specific for H2B and H3 combined with RNAi-mediated protein depletion. Complementing the FAIRE data, depletion of BAF250 and BRG1, but not BAF200 or BAF180, caused a strong loss of histone H2B and H3 at nuc-1, which is concomitant with increased DNA accessibility at this position (Fig 6C and S6C Fig). Interestingly, loss of histone density at nuc-1 was accompanied by an increase in histone density within the DHS1 region (Fig 6C and S6C Fig).

To examine if knock-down of individual subunits affects recruitment of the complexes to the LTR, we performed ChIPs to monitor BRG-1 enrichment at the LTR in BAF180 or BAF250-depleted J-Lat 11.1 cells in the latent versus PMA stimulated states (Fig 6D). We found that depletion of BAF250 abrogated complex recruitment to the LTR in the latent state but had no effect on recruitment of PBAF to nuc-1 in response to PMA stimulation. Vice versa, BAF180 depletion abrogated PMA dependent recruitment of the BRG-1 complex to nuc-1, but had no effect on the enrichment of BAF at the LTR nuc-1 in the latent state (Fig 6D). Thus, while BAF- and PBAF-specific factors appear to be targeting subunits required for complex recruitment to the LTR, they do not affect the recruitment of the other functionally distinct complex to the LTR. Together our data indicate that while PBAF is recruited by Tat to the HIV LTR and required for Tat-mediated activation, the BAF complex is essential for repression of basal LTR activity by countering histone-DNA sequence preferences at the LTR and positioning the repressive nuc-1 over less optimal sequences immediately downstream of the transcription start site.

### High Resolution Nucleosomal Mapping Reveals a Dramatically Altered LTR Chromatin Structure After Depletion of BAF250

**Fig 7:**
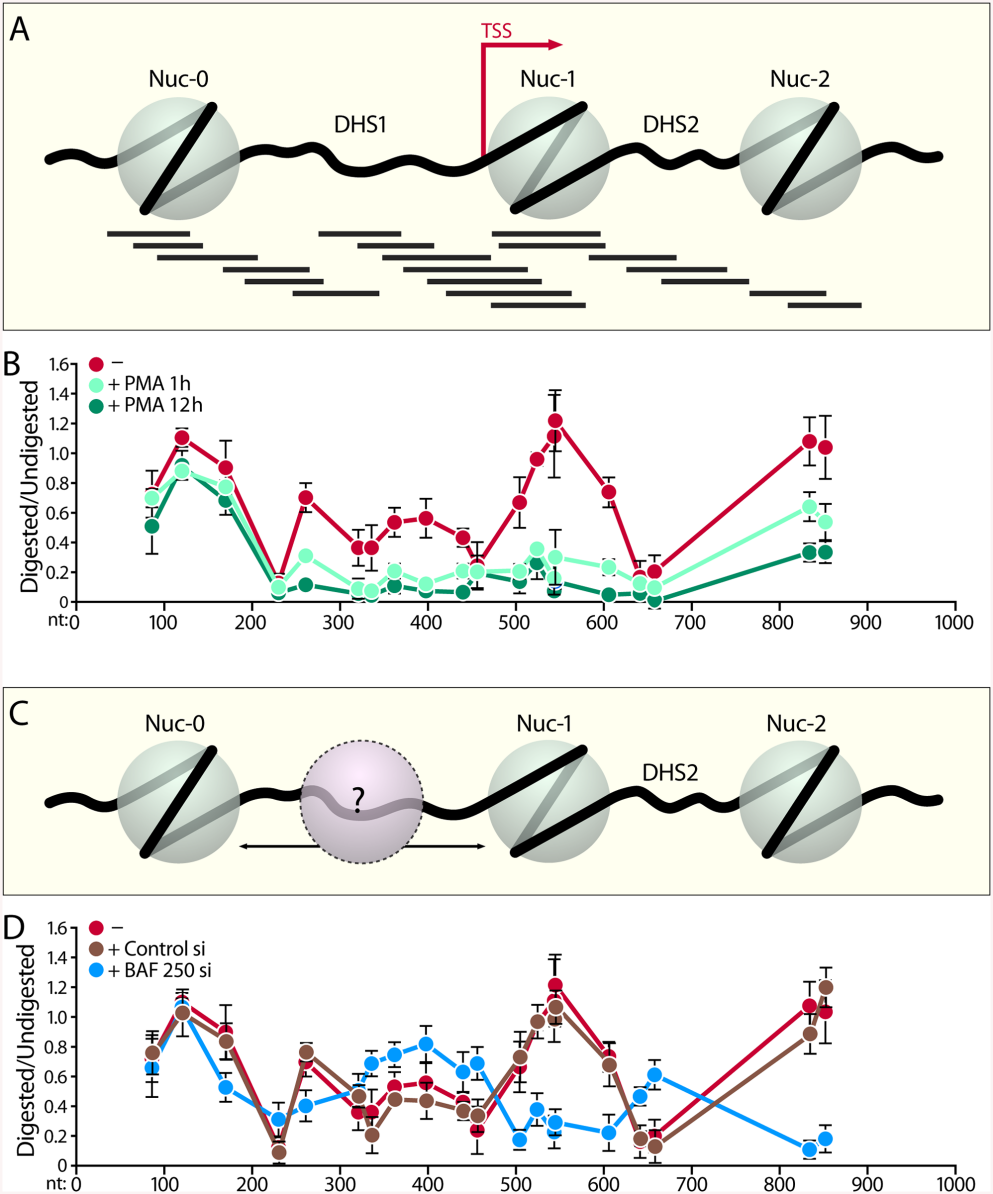
BAF250 depletion induces change in chromatin structure at the HIV LTR as detected by High Resolution MNase nucleosomal mapping. (A) Diagram showing the PCR amplicons used at the HIV LTR covering nucleotides 40–902 corresponding to Nuc0, DHS1, Nuc-1, DHS2, and Nuc-2. PCR products are 100 ± 10 bp in size and are spaced approximately 30 bp apart. (B) Change in chromatin structure of the HIV LTR in J-Lat 11.1 cells upon PMA stimulation. The chromatin profile of the HIV LTR was determined at 0 (red line), 1 h (light green line), and 12 h (dark green line) post-PMA stimulation by normalizing the amount of the MNase digested PCR product to that of the undigested product using the ΔC(t) method (*y*-axis), which is plotted against the midpoint of the corresponding PCR amplicon (*x*-axis). The *x*-axis represents base pair units with 0 as the start of LTR Nuc-0. Error bars represent the average of three independent experiments. (C) A loosely positioned nucleosome between the positioned Nuc-0 and nuc-1 is indicated in pink. (D) Depletion of BAF250 induces restructuring of the HIV LTR chromatin profile. The chromatin profile of the HIV LTR was determined in J-Lat 11.1 cells nucleofected with either control siRNA (brown line) or siRNA targeting BAF250 (blue line). The chromatin profile of untransfected cells are also provided for comparison (red line). Error bars represent the SEM of three independent experiments.

Our data thus far demonstrated a critical role for the BAF250a containing BAF chromatin remodeling complex in actively maintaining a repressive nucleosomal structure at the HIV LTR. To obtain a higher resolution picture of the dynamic changes in LTR nucleosomal structure in the presence or absence of BAF250, we performed high resolution MNase nucleosomal mapping, as previously described [59,60]. We made slight modifications to this protocol to be able to reproducibly visualize in vivo changes in the LTR chromatin structure in the lower numbers of cells obtainable after depletion of BAF250 by siRNA nucelofection. After formaldehyde cross-linking, the isolated chromatin was divided into undigested and MNase digested samples. Digested and undigested DNA was then probed with 20 separate overlapping primer sets, amplifying approximately 100 bp regions along the HIV LTR (Fig 7A and S1 Table). MNase cleaves nucleosome-free and linker DNA connecting two nucleosomes, while DNA within nucleosomes is at least partially protected and resistant to digestion. Therefore, the amount of DNA remaining between two primers after MNase digestion, which determines the ability of a primer pair to amplify that region by real-time qPCR, can be used to show the amount of digestion in a particular amplicon at the HIV LTR. We calculated the relative ratio of the amount of digested DNA to the undigested control for each overlapping primer pair scanning the length of the HIV LTR (Fig 7A and 7B).

We examined first the LTR nucleosomal profile in J-lat 11.1 cells which were either unstimulated or treated with PMA for 1 or 12 h (Fig 7B red line). As shown previously [5–7], we find that under unstimulated conditions, the HIV LTR contains two distinct chromatin regions: a nucleosome positioned immediately after the TSS (Nuc-1), and a second nucleosome (Nuc-0) at the 5′ end of the LTR. 3′ to the LTR, there is another strictly positioned nucleosome (Nuc-2) as well as an intervening MNase hypersensitive DNA region between the positioned Nuc-1 and Nuc-2. Surprisingly, we found that DHS1 (nucleotides 200–452) connecting Nuc-0 and Nuc-1 was not devoid of nucleosomes, but rather contained poorly positioned nucleosomes as determined by partial protection from MNase digestion (Fig 7B and 7C). This area, previously demonstrated to be hypersensitive to nuclease digestion, contains consensus binding sites for a range of host cell transcription factors critical for LTR activity. PMA stimulation at 1 h caused a striking decrease in DNA protection between the positioned Nuc-0 and Nuc-2, indicating a loss of nucleosomes within this region including the positioned Nuc-1 (Fig 7B, light green line). At 12 h of PMA stimulation, a more significant and broader loss of nucleosomal DNA protection occurred downstream of the positioned Nuc-0, which now included the positioned Nuc-2 (Fig 7B, dark green line).

We next examined what effect depletion of BAF250a has on the observed high resolution LTR nucleosomal structure. We depleted BAF250 from J-Lat 11.1 cells using siRNA transfection and compared the resulting LTR nucleosomal profile to that of cells transfected with control siRNA (Fig 7D). As expected, nucleofection of cells with control siRNA had no effect on the LTR nucleosomal structure (Fig 7D, red and brown lines). However, siRNA depletion of BAF250 resulted in a dramatically altered LTR chromatin structure (Fig 7D, blue line). Confirming our results from FAIRE experiments, depletion of BAF250 coincided with a loss of the strictly positioned Nuc-1. Interestingly, the sequences within the previously described DHS1 (between Nuc-0 and Nuc-1) and DHS2 (between Nuc1 and Nuc-2) showed significantly more resistance to MNase digestion in the BAF250-depleted samples. Thus, the nucleosomal landscape of BAF250a-depleted J-Lat cells more closely resembles the predicted nucleosome positioning within the LTR (Fig 6A), which is determined by intrinsic histone-DNA sequence preferences. Thus, confirming and extending our data obtained from FAIRE and histone ChIP experiments (Fig 6 and S6 Fig), the observed changes in the high resolution MNase chromatin profile of the LTR in response to BAF250a depletion demonstrates a loss of histones within Nuc-1 concomitant with an increase in histone density over the DHS1.

### Depletion of BAF Decreases the Incidence of Latent HIV Infections in Jurkat and SupT1 Cells

**Fig 8:**
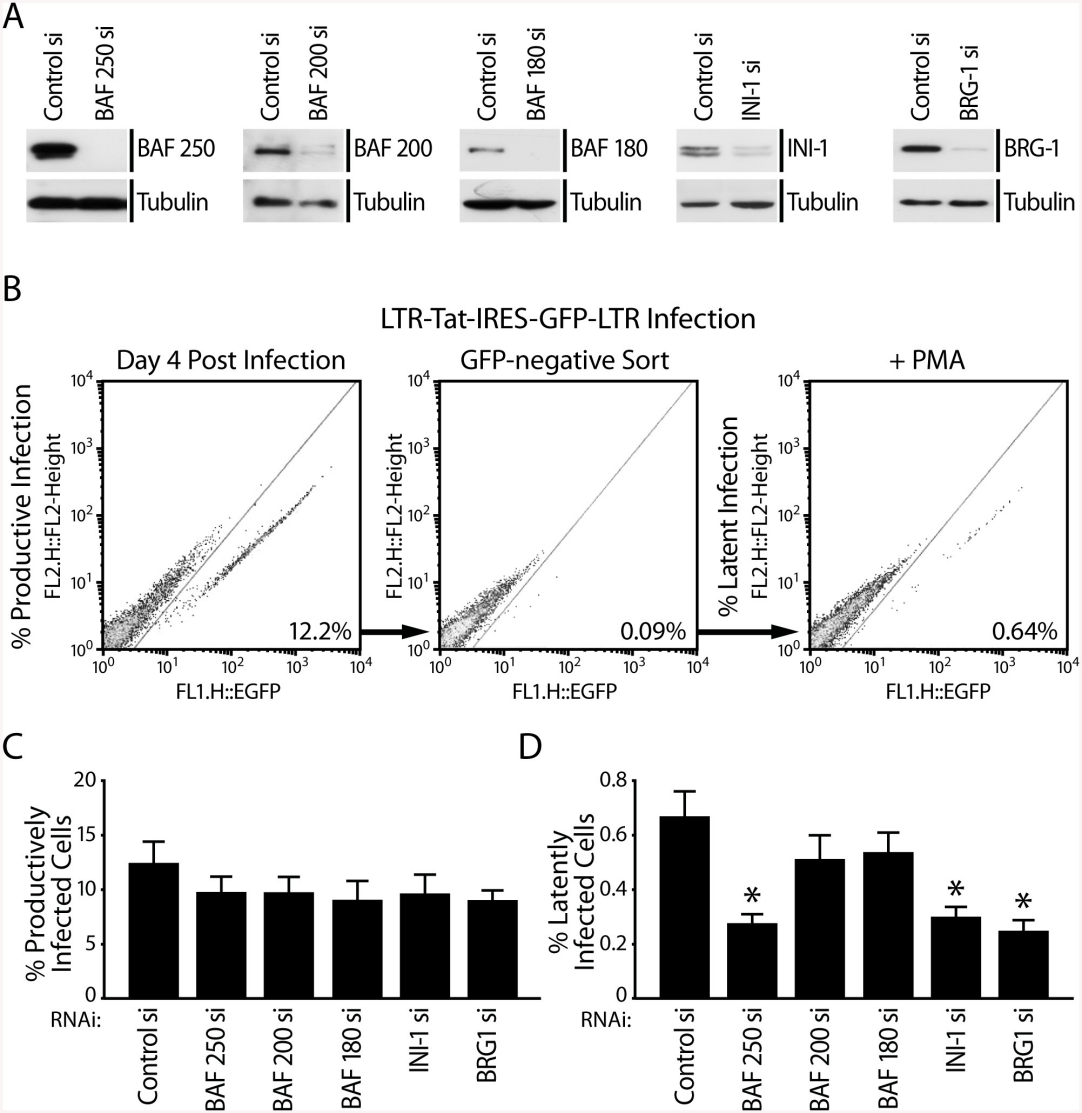
BAF promotes the establishment of latent HIV infections. (A) Western blot analysis demonstrates depletion of BAF/PBAF subunits as indicated 96 h after siRNA transfection. (B) Schematic representation of protocol for comparison of productive and latent infections in Jurkat cells containing or depleted of remodeling complex subunits. Jurkat cells were first nucleofected with control siRNA or siRNA targeting individual SWI/SNF subunits as indicated. After 48 h, cells were infected with retroviral particles containing the vector LTR-Tat-IRES-GFP. The percentage of productive infections (GFP-positive cells obtained after infection (12.2%) (left panel)), FACS-sorted GFP-negative cells (middle panel), or percent latent infections (GFP-positive cells obtained after PMA treatment of sorted GFP-negative cells (0.64%) (right panel)) are shown for Jurkat cells nucleofected with control siRNA. (C) Depletion of BAF/PBAF subunits as indicated does not significantly affect the percentage of productive HIV infections. (D) Depletion of the core SWI/SNF subunits BRG1 and INI-1 and the BAF-specific subunit BAF250 significantly decreases the incidence of latent HIV infections (the percent GFP positive cells obtained after PMA stimulation of GFP negative cell population) while depletion of PBAF-specific subunits BAF200 or BAF180 had no significant effect on latency establishment.

The de-repression of latent infections observed in J-LatA2 and J-Lat 11.1 cells in response to depletion of the BAF complex subunits BAF250, INI-1, and BRG1 indicated that the BAF complex is necessary to maintain silencing at the LTR during a latent infection (Fig 3). Since depletion of BAF resulted in the loss of the positioned repressive nuc-1 (Fig 6), we wondered whether BAF contributes to the establishment of latent HIV infections by positioning nuc-1 and repressing basal HIV transcription. We therefore tested whether depletion of BAF would decrease the establishment of latent infections. We used siRNAs to deplete BAF250, or the core subunits BRG1 or INI-1, or the PBAF-specific subunits BAF200 and BAF180 from Jurkat cells (Fig 8A). In parallel we examined the effect of BAF/PBAF depletion in latency establishment in another CD4+T cell line, SupT1 cells (S7 Fig). Using a strategy we and others described previously [46,61], we then compared the percentage of latent HIV infections established in the presence or absence of BAF/PBAF subunits (Fig 8B and S7A Fig). Briefly, we infected Jurkat or SupT1 cells at low multiplicity of infection with an HIV-1-derived virus containing a GFP reporter, LTR-Tat-IRES-GFP. Percent productive infections were scored as percent GFP positive cells 72 h after infection with virus (Fig 8B and 8C, S7A and S7B Fig). Using Flow Cytometry (FACS) we sorted the GFP negative population, which presumably contained uninfected as well as latently infected cells. Treatment of the GFP negative population with PMA led to activation and GFP expression of the latently infected population, which was analyzed and quantitated by FACS (Fig 8B and 8D, S7A and S7C Fig). As shown in Fig 8C and S7B Fig, the percentage of productive infections was slightly decreased in the absence of BAF/PBAF subunits. However, depletion of the core subunits INI-1 or BRG1 or the BAF-specific subunit BAF250 resulted in a significant, greater than 50% lower incidence of latent infections in both Jurkat and SupT1 cells, while depletion of PBAF-specific subunits had no significant effect on latency establishment (Fig 8D and S7C Fig). We also tested whether similar to BAF, depletion of CHD3 also decreases the incidence of latent infections (S8 Fig). Using siRNAs we depleted CHD3, BAF250, and BRG-1 either individually or simultaneously in both Jurkat and SupT1 cells (S8 Fig). Similar to BAF250 and BRG-1 depletion, depletion of CHD3 resulted in decreased incidence of latent infections. However, we found no synergistic effect on latency establishment when both BAF250 and CHD3 or BRG1 and CHD3 were simultaneously depleted by siRNA transfection. These results suggest that the BAF complex contributes to the establishment of latent infections and points to the ATP-dependent enzyme BRG1 as a putative therapeutic target to deplete the latent HIV-infected reservoir in infected patients.

## Discussion

**Fig 9:**
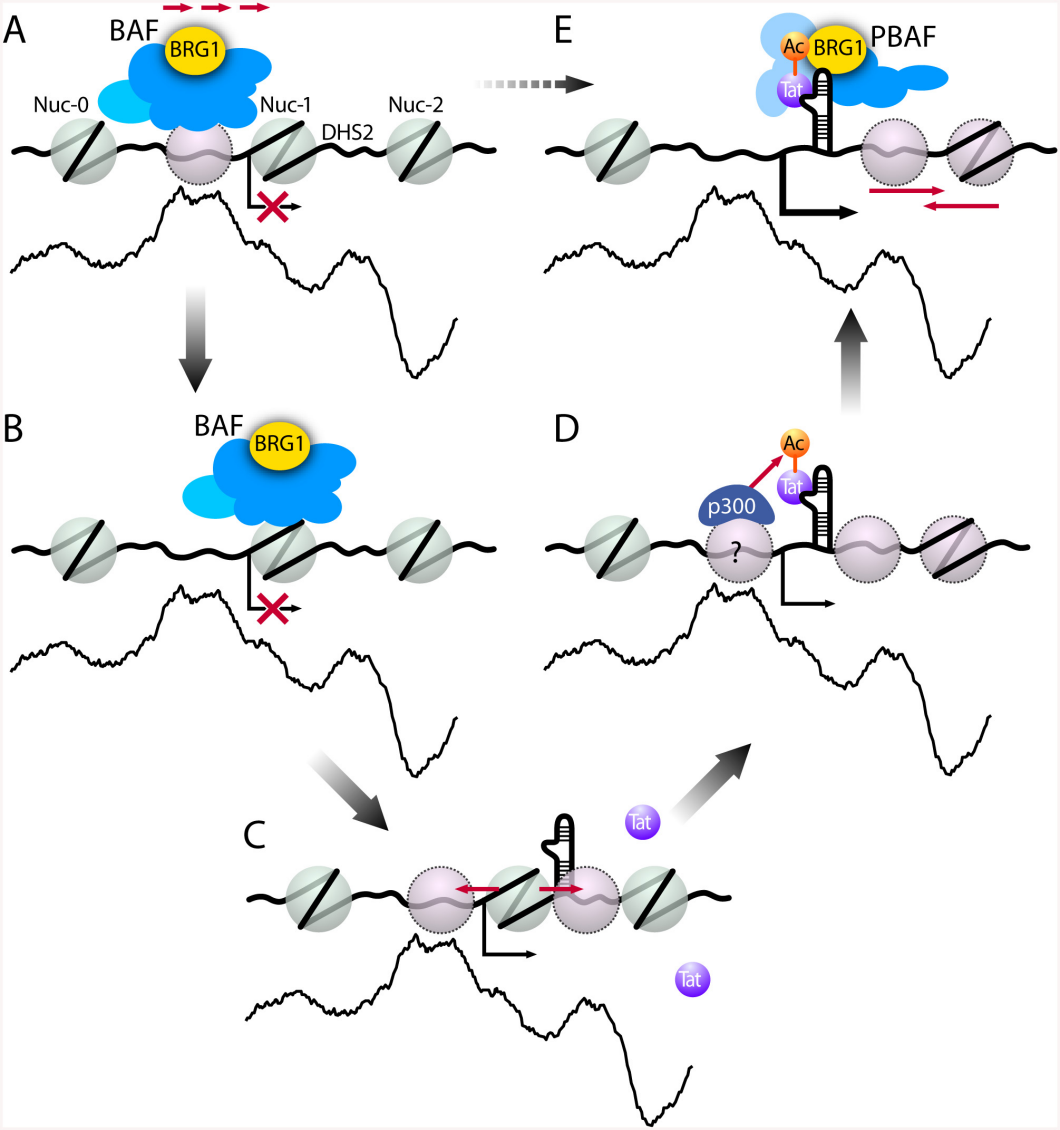
Model for SWI/SNF regulation of HIV LTR transcription. BAF uses energy from ATP hydrolysis to actively counteract intrinsic histone-DNA sequence preferences within HIV LTR. (A–B) BAF pulls/pushes a preferred nucleosome over DHS1 onto DNA sequences less favorable for nucleosome formation immediately downstream of the transcription start site (TSS), leading to positioning of nuc-1 and transcriptional repression. (C) Upon activation, BAF dissociates from the LTR resulting in re-positioning of the nucleosomes to thermodynamically more favorable positions leading to de-repression of HIV transcription. (D) Upon Tat expression, p300, recruited to the LTR, acetylates Tat. (E) p300-acetylated Tat then selectively recruits the PBAF complex, which uses energy from ATP hydrolysis to actively re-position nucleosomes formed downstream of TSS enabling efficient transcription elongation.

Our results suggest a novel model for the regulation of HIV transcription and the role of the SWI/SNF chromatin-remodeling complex (Fig 9). We find that, on the HIV LTR, active chromatin remodeling is required for the generation of a chromatin conformation that is repressive to transcription; the BAF complex strictly positions nuc-1. Thus, in the absence of Tat, BAF is bound to the HIV promoter where it represses transcription by counteracting intrinsic nucleosome-DNA sequence preferences and positioning nuc-1 in a less energetically favorable position immediately downstream of the TSS. Upon activation, BAF is removed from the LTR, allowing the formation of nucleosomes according to their intrinsic histone-DNA sequence preferences. Upon expression, Tat first recruits acetyltransferases resulting in the acetylation of promoter histones as well as Tat itself [62–66]. Intriguingly, another SWI/SNF complex, PBAF, specifically interacts with acetylated Tat and is recruited to the HIV promoter by Tat in vivo. Thus, the biochemically distinct chromatin-remodeling complexes BAF and PBAF display functional specificity on the HIV promoter, one repressive and the other participating in the transcriptional activation of the HIV promoter by Tat. This model presents a considerably more complex picture of the role of SWI/SNF proteins in the transcriptional regulation of HIV expression than had previously been anticipated.

The involvement of the BAF complex in repressing basal HIV transcription raises a number of novel questions. Intriguingly, HIV-1 integrase interacts with the SWI/SNF protein INI-1 in vitro and in vivo [41]. During HIV infection, incoming retroviral pre-integration complexes trigger the cytoplasmic export of the SWI/SNF component INI1 and of the nuclear body constituent PML [50]. The HIV genome associates with these proteins before nuclear migration. In the presence of arsenic, PML is sequestered in the nucleus and the INI-1/preintegration complex interaction is disrupted [50]. Under these conditions, the efficiency of HIV-mediated transduction is markedly increased. This observation could be explained in part by our observations that the BAF complex represses basal HIV transcription by positioning the repressive nuc-1.

Why would the HIV virus position a repressive nucleosome immediately downstream of its transcriptional start site? An attractive possibility is that BAF may remove or pull the thermodynamically favored nucleosome away from the promoter (DHS1) to allow for binding of host cell transcription factors whose binding sites are present within the DHS1. In particular, the NFKB and Sp1 consensus sites within the DHS1 have been shown to be critical for basal HIV LTR promoter activity [67]. In this context, positioning of nuc-1 by BAF downstream of the TSS would make consensus sites within the DHS1 accessible for binding by sequence-specific transcription factors, and allow for the assembly of the initiation complex. Surprisingly, our high-resolution MNase nucleosomal mapping of the LTR demonstrated that DHS1 is not devoid of nucleosomes as previously implied. The DNA within this region displayed partial protection against MNase digestion, indicating the presence of loosely positioned nucleosomes, which disappeared upon PMA stimulation. Interestingly, a previous study by Workman and colleagues argued for the presence of a nucleosome over the HIV promoter (DHS1) and the formation of a ternary complex consisting of the transcription factors, histones, and DNA [68]. It is important to note that in this study the HIV promoter/DHS1 sequence was reconstituted into mono-nucleosomes or placed within an array of positioned nucleosomes in vitro and outside the context of the adjacent nuc-0 and nuc-1 sequences [68]. Our modeling data, depicting the high predicted affinity (nucelosome score) of the DHS1 DNA sequence (Fig 6A), are in agreement with these observations implying the presence of a nucleosome over the DHS1 region in vitro.

Our high-resolution MNase nucleosomal mapping data provide a detailed picture of the dynamic nucleosomal landscape of the HIV LTR, comparing the latent, PMA activated, and BAF-depleted de-repressed LTR states. Loss of BAF250a caused a dramatic re-positioning of the nucleosomes according to their intrinsic DNA-histone sequence preference; DNA encompassing nuc-1 became hypersensitive and susceptible to digestion by MNase while the DHS sites were rendered less accessible and protected from MNase, more closely resembling the predicted LTR nucleosomal structure (Fig 6A). These results support a nucleosome repositioning model upon BAF250a depletion, which contrasts with the observed eviction of nucleosomes downstream of the positioned Nuc-0, which occurs after PMA stimulation.

An important question remaining to be resolved is how BAF is recruited to the LTR to position nuc-1. SWI/SNF complexes have been shown to function gene-specifically, recruited by sequence-specific transcription factors to regulatory regions of target genes. The HIV LTR contains many binding sites for multiple sequence-specific host transcription factors, including SP1, NFkb, YY-1, NFAT, LBP1, etc. One possible mechanism is the recruitment of BAF by a repressive sequence-specific transcription factor bound to nuc-1. A number of transcriptional repressors contain binding sites within the region occupied by nuc-1, including LSF-1 and YY-1 [69]. Indeed, the transcriptional repressor YY-1 was bound to the HIV promoter under basal conditions and was displaced in response to Tat expression [38]. Thus, YY-1 is a candidate transcription factor, which may recruit BAF to the HIV LTR to position the repressive nuc-1. In support of this possibility, pleiohomeotic (PHO), the Drosophila homologue of YY-1, has been shown to directly recruit the SWI/SNF complex to target genes [70]. Conversely, the BAF complex, which itself may be recruited by another sequence-specific transcription factor, may allow for YY-1 binding, leading to de-acetylation of histones at the LTR, and the repression of HIV transcription under basal conditions.

Our results indicate that BAF-remodeling activity is necessary to position nuc-1 downstream of the transcriptional start site. The fact that nuc-1 becomes remodeled upon ATP depletion is consistent with this model and, within the limitations of this experiment, suggests that nuc-1 positioning downstream of the TSS is an active process driven in part by ATP and BAF activity. Complementing the ChIPs, we used FAIRE to assess changes in DNA accessibility within nuc-0, DHS1, and nuc-1 regions of the HIV LTR in response to specific depletion of BAF or PBAF. The mapping of accessible DNA within the LTR by FAIRE negatively correlated with histone H3 and H2B occupancy. Our data indicate that the specific depletion of BAF causes nucleosome repositioning in accordance with intrinsic histone-DNA sequence preferences; in the absence of BAF, the DHS1 region, whose sequence displays a higher propensity for nucleosome formation, displays higher histone density and lower accessibility, while the opposite profile is observed for the DNA sequence encompassing nuc-1. These observations suggest that the energetic cost of positioning the repressive nuc-1 downstream of the TSS is provided and driven by ATP hydrolysis. BAF positions nuc-1 by either pushing the nucleosome from its optimal sequence over DHS1 or by pulling it onto sub-optimal sequences encompassing nuc-1. Thus, the BAF complex counteracts and overrules the DNA sequence effects and intrinsically favored nucleosome position over the DHS1 region of the HIV LTR.

The dramatic increase in DNA accessibility over nuc-1 concomitant with de-repression of LTR activity observed upon depletion of the BAF complex begs the following question: why is recruitment of PBAF by Tat necessary to drive transcription at the LTR? In such a context, the recruitment by Tat of an alternative complex, PBAF, may be necessary for remodeling of the nucleosomes, formed in the absence of BAF according to their preferred DNA sequences, downstream of the TSS, leading to efficient transcription elongation (Fig 9). Indeed, BRG1 was recently shown to be recruited to and facilitate RNA Pol II to overcome nucleosomal barriers during transcription elongation in vivo [71].

The balance between activating and repressive cofactors at the LTR is believed to determine the level of basal transcription from the LTR in the immediate early, Tat-independent phase of HIV transcription. The local availability of positive and negatively acting cofactors at the LTR therefore determines the likelihood of transcriptional silencing. Our data demonstrated a critical role for BAF in positioning nuc-1 and maintaining HIV LTR silencing. We found that depletion of BAF led to a de-repression of latent HIV in the J-Lat system reflecting HIV latency. Despite the effectiveness of modern HAART regimens, latent HIV-infected cells persist in patients, providing the main impediment to cure from HIV infection. Recently, the need for the development of new strategies to treat HIV-infected patients has been discussed [72], highlighting the necessity to deplete the latent HIV-infected reservoirs. As the catalytic subunit of the BAF complex, the enzyme BRG1 may present an attractive candidate for drug targeting to purge the latent HIV-infected reservoir in the treatment of HIV.

## Materials and Methods

### Cell Lines and Plasmids

We used the following cell lines: Jurkat clones D and E (containing integrated LTR-GFP), J-Lat A2 (integrated latent LTR-Tat-IRES GFP), J-Lat 11.1 (integrated latent full-length HIV genome containing a mutation in the env gene and GFP in place of the nef gene) [45,46], and Jurkat 1G5 cells containing integrated LTR-Luciferase [54]. The HIV LTR-luciferase reporter construct (pEV229), the CMV-driven expression vectors for FLAG-tagged wild-type Tat (pEV280), FLAG-tagged mutant Tat(K50R/K51R) (pEV538), and p300 have been described [62]. Plasmids used to generate HIV-derived virus particles, vesicular stomatitis virus envelope (VSVG), the NL4-3 packaging vector (R8.91), and the retroviral vector LTR-Tat-IRES-EGFP (pEV731), have been previously described [45].

### Sodium Azide Treatment and Chromatin-Remodeling Assay

Exponentially growing Jurkat cell line D or J-Lat A2 were treated with increasing concentrations of sodium azide overnight. Restriction enzyme accessibility with AflII was performed on intact nuclei followed by Southern blotting as previously described [45]. Briefly, cells were harvested by centrifugation and washed with ice-cold PBS. The subsequent steps were performed on ice with precooled buffers. Cells (10^7^) were resuspended in 400 μl of buffer A (10 mM Tris, pH 7.4, 10 mM NaCl, 3 mM MgCl_2_, 0.3 M sucrose) and incubated on ice for 10 min. An equal volume of buffer A/0.2% NP-40 was added, and cells were incubated for a further 10 min. Nuclei were pelleted at 240×g for 10 min, resuspended in 50 μl of buffer B (10 mM Tris, pH 7.9, 10 mM MgCl_2_, 50 mM NaCl, 1 mM dithiothreitol, 100 μg/ml BSA, 0.1 mM phenylmethylsulfonyl fluoride), and digested for 20 min with AflII (0.5 U/μl) at 37°C. Digestion reactions were placed on ice, and genomic DNA was purified with DNeasy Tissue kit (Qiagen). The same amounts of DNA from each sample were digested to completion with NcoI. The extent of AflII cleavage was detected by southern blotting. Hybridization was performed with a ^32^P-labeled PCR probe corresponding to a fragment internal to the 5′LTR generated from pRRL GFP vector by PCR. Primer sequences are provided in S1 Table.

### Antibodies, Coimmunoprecipitation, and Western Blot Analysis

Anti-BRM, anti-SMARCB1 and anti-SMARCA5/hISWI (Abcam), anti-SMARCA4/BRG1, anti-ARID1a/BAF250a and anti-14-3-3 (Santa Cruz Biotechnology), anti-BAF200 (kind gift from C.P. Verrijzer), and anti-BAF180 (abcam and kind gift from W. Wang and D. Murray) were used in Western blot and immunoprecipitation experiments. For immunoprecipitations, Jlat A2 cells were treated with 10 nM phorbal 12 myristate 13-acetate (PMA) for 12–16 h to produce Tat-FLAG. Cells were lysed in IP buffer (25 mM HEPES, pH 7.9, 150 mM KCl, 1 mM EDTA, 5 mM MgCl_2_, 5% glycerol, 1% NP40, 0.5 mM dithiothreitol, 1 uM TSA, 1 mM nicotinamide, and a protease inhibitor (PI) cocktail (Sigma)) for 20 min on ice and passed through a 26-gauge needle twice. Lysates were centrifuged, and 2 mg of whole-cell protein lysate was incubated with 20 ul M2 agarose beads (Sigma) in immunoprecipitation (IP) buffer overnight at 4°C on a rotator. After five washes with IP buffer, beads were resuspended in SDS loading buffer, and co-immunoprecipitated proteins were separated on an SDS-PAGE gel and identified by Western blotting. For Tat immunoprecipitation in 293T cells, cells were transfected with empty pcDNA3.1 or N-terminally FLAG-tagged wild-type or mutant K50R/K51R Tat (pEV280 or pEV537) in presence or absence of expression vector for p300; 36 h after transfection, cells were stimulated with 1 μm TSA and 5 mm nicotinamide for 6 h, harvested, and lysed in buffer (phosphate-buffered saline, 2 mm EDTA, 1% Triton X-100, 0.5 mm dithiothreitol, 1 μm TSA, 5 mm nicotinamide and PI cocktail). 5 mg protein lysate was incubated overnight with 40 μl M2-agarose beads at 4°C on a rotator. Beads were washed extensively with lysis buffer, resuspended in SDS loading buffer and co-immunoprecipitaed proteins identified by Western blot analysis using the indicated antibodies.

### Amaxa Nucleofection and siRNA Depletion

Nucleofection of Jurkat cells, SupT1 cells, and Jurkat cell clones D, E, J-Lat A2, 11.1, and 1G5 cells was conducted as previously described [38]. Cells were split to 3×10^5^ cells/ml 24 h before Amaxa nucleofection. Five million cells were centrifuged at 1,000 rpm for 10 min at room temperature, resuspended in 100 μl of solution R, and nucleofected with 20 nM siRNA or 2 μg of expression plasmid using program O28. Nucleofected cells were resuspended in 500 μl of prewarmed, serum-free RPMI lacking antibiotics and allowed to recover at 37°C in a 5% CO_2_ incubator for 15 min. Prewarmed complete RPMI (4 ml) was then added to the cells. Dharmacon siRNA control and on-target smartpools targeting transcripts of the human SMARCB1, SMARCA4, SMARCA2, SMARCA5, CHD3, INO80, PB1, ARID2, ARID1a, and ARID1b genes were used to knockdown the expression of respective genes in Jurkat and SupT1 cells, Jurkat clones D, E, J-Lat A2, 11.1, and 1G5 cells. Protein levels were examined by Western blot analysis 0, 2, 4, 6, 7, 8, 10, and 14 d after nucleofection.

### Flow Cytometry

Samples were analyzed on a FACS Calibur flow cytometer with Cell Quest software (Becton Dickinson). The live population was defined by forward versus side scatter profiles. Cells were further gated by using forward scatter versus FL1 to differentiate between GFP-positive and -negative cells. GFP expression in the J-Lat cell lines was analyzed by FACS at 0, 2, 4, 6, 7, 8, 10, 12, and 14 d after siRNA nucleofection.

### Chromatin Immunoprecipitation, FAIRE, and Quantitative PCR (qPCR)

J-Lat A2 and 11.1 cells were fixed by adding formaldehyde to a final concentration of 1% for 10 min for histone IPs and FAIRE and 30 min for BAF/PBAF subunit IPs at RT. The reaction was quenched with 125 mM glycine, cells washed with buffer B (0.25% Triton-X 100, 1 mM EDTA, 0.5 mM EGTA, 20 mM Hepes, pH 7.6), buffer C (150 mM NaCl, 1 mM EDTA, 0.5 mM EGTA, 20 mM Hepes, pH 7.6), and resuspended in ChIP incubation buffer (0.3% SDS, 1% Triton-X 100, 0.15 M NaCl, 1 mM EDTA, 0.5 mM EGTA, 20 mM Hepes, pH 7.6). Chromatin was sheared by sonication to an apparent length of ~200–400 bp (corresponding to ~100–200 bp of free DNA) using a BioRuptor sonicator (Cosmo Bio Co., Ltd) with 22 45-s pulses at maximum setting. More than 20 million cells were used per IP, and 5 μg of the indicated antibody was incubated with the chromatin and BSA-blocked protein G beads overnight at 4°C. IPs were washed twice with each buffer 1 (0.1% SDS, 0.1% deoxycholate, 1% Triton-X 100, 150 mM NaCl, 1 mM EDTA, 0.5 mM EGTA, 20 mM Hepes pH 7.6), buffer 2 (0.1% SDS, 0.1% deoxycholate, 1% Triton-X 100, 0.5 M NaCl, 1 mM EDTA, 0.5 mM EGTA, 20 mM Hepes pH 7.6), buffer 3 (250 mM LiCl, 0.5% deoxycholate, 0.5% NP-40, 1 mM EDTA, 0.5 mM EGTA, 20 mM Hepes, pH 7.6), and buffer 4 (1 mM EDTA, 0.5 mM EGTA, 20 mM Hepes, pH 7.6). Immunoprecipitated complexes were eluted in elution buffer (1% SDS, 0.1 M NaHCO_3_) for 20 min at RT, and decrosslinked overnight at 65°C in presence of 200 mM NaCl_2_. DNA was phenol:chloroform extracted, chroloform:isoamylalcohol extracted, ethanol precipitated, and resuspended in 100 ml H_2_O by shaking at 37°C. Input and immunoprecipitated DNA (5 μl) were subjected to Sybergreen Q PCR cycles with specific primers. For FAIRE, cells were subjected to formaldehyde crosslinking for 10 min. 20 mg of cross-linked chromatin was diluted 9× with buffer D (150 mM NaCl, 20 mM Tris-HCl pH 8.1, 2 mM EDTA pH8.0, 1% Triton-X100 and PI cocktail) and phenol-chloroform extracted. Isolated DNA was subjected to Sybergreen Q PCR cycles with specific primers.

### High- Resolution MNase Nucleosomal Mapping

High resolution MNase mapping protocol [59,60] of the HIV LTR was slightly modified to be amenable to the lower cell numbers obtainable after siRNA depletion of specific factors. Briefly, cells were cross-linked according to the ChIP protocol described above. After one wash in cold PBS, 1.5 x 10^7^ cross- linked cells were resuspended in 1 ml hypotonic buffer A (300 mM sucrose, 2 mM Mg acetate, 3 mM Cacl2, 10 mM Tris pH 8.0, 0.1% Triton X-100, 0.5 mM DTT), incubated on ice for 5 min, and dounced 20 times with 2 ml dounce homogenizer (tight pestle, Wheaton). Nuclei were collected by centrifuging at 4°C for 5 min at 720 x g. Pellets were resuspended in 1 ml buffer D (25% glycerol, 5 mM Mg acetate, 50 mM Tris pH 8.0, 0.1 mM EDTA, 5 mM DTT) at 1.5 x 10^7^ nuclei/ml. Chromatin was collected by centrifuging at 4°C for 5 min at 720xg. The pellets were resuspended in 1 ml buffer MN (60 mM KCl, 15 mM NaCl, 15 mM Tris pH 7.4, 0.5 mM DTT, 0.25 mM sucrose, 1.0 mM CaCl2) at 2.5 x 10^7^ nuclei/ml. The equivalent of 2.5 x 10^6^ nuclei were used per MNase reaction. MNase (USB), diluted in buffer MN, was added so that 0, 0.5, 5, 20, 50, and 500 total units were used per 150 ul reaction and digested for 30 min at room temperature. Reactions were stopped with the addition of EDTA and SDS to final concentrations of 12.5 mM and 0.5% respectively. After 4 hours of proteinase K digestion at 37°C, each reaction was processed similar to ChIP samples from the point of elution from the beads.

### Real-Time qPCR Analysis

ChIP, FAIRE, and MNase digested samples were analyzed by quantitative PCR in an iCycler iQ real-time PCR detection system (BioRad) using iQ Sybergreen Supermix (BioRad). ChIP values were normalized as a percentage of input. Sequences of qPCR primer pairs used to amplify distinct regions within the HIV-1 LTR are provided in S1 Table. For MNase digests a fold difference was calculated using the ΔCT method between MNase treated and untreated samples. All values used were collected from the linear range of amplification.

### Analysis of HIV LTR Sequence for Nucleosome Propensity

The NuPoP algorithm has been described [55]. An alternative algorithm was also used to predict the nucleosome affinity for HIV nucleotide sequence 1–1800 [56,57]. Estimates of the genomic sites of HIV integration were derived from [58].

### Latency Establishment Experiment

HIV-derived virus particles were generated as described [45]. Briefly, 293T cells were transfected with VSVG, the NL4-3 packaging vector, and the retroviral vector LTR-Tat-IRES-EGFP (pEV731). Virus was harvested every 12 h starting at 24 h after transfection. Jurkat or SupT1 cells containing or depleted of BRG1, BAF250, BAF200, BAF180, INI-1, or CHD3, by siRNA transfection, were infected with the LTR-Tat-IRES-EGFP virus at low MOI such that less than 20% of cells were infected. 96 h after infection, the GFP negative cell population harboring uninfected as well as presumably latently infected cells were sorted (once or twice depending on the purity of the GFP negative population) by Flow Cytometry Activated Cell sorting (FACS). GFP negative cells were then treated with PMA and analyzed by FACS after 24 h to determine the percent GFP positive (latent) infections.

## Supporting Information

S1 Fig ATP depletion results in nuc-1 remodeling and HIV promoter activation. (A) Schematic representation of the restriction sites and probe used to analyze the remodeling of nuc-1. Nuclei isolated from cells treated either with PMA or sodium azide (NaN_3_) were digested in vitro with AflII to probe for accessibility of the DNA encompassing nuc-1. Genomic DNA was subsequently digested with *NcoI* in vitro, and the DNA was analyzed by indirect-end labeling. The *NcoI* genomic fragment (fragment B) and the double *NcoI/AflII* digestion product (fragment A) are shown. (B) Indirect-end labeling after PMA or NaN_3_ treatment and (C) corresponding increase in GFP expression in Jurkat clone D containing an integrated LTR-GFP virus. (D) Indirect-end labeling after NaN_3_ treatment and (E) corresponding increase in GFP expression in J-Lat A2 containing an integrated latent LTR-Tat-IRES-GFP virus. GFP, measured by flow cytometry, is shown as mean fluorescence intensity (MFI) (C) or increase in percent GFP positive cells (E) 16 h after treatment as detailed above. The intensities of bands from three experiments were quantitated using Odyssey software and used to compare fold increase in ratio of bands A/B in each condition and plotted as mean ± SEM. * *p*<0.05, ** *p*<0.01.
(TIF)

S2 Fig Stability of SWI/SNF complex component protein levels after siRNA depletion of individual subunits. Jurkat cells containing an integrated LTR-GFP virus (clone D) were transfected with either control siRNA or siRNAs targeting various SWI/SNF complex subunits as indicated. Western blot analysis shows expression of SWI/SNF complex subunits in presence of siRNA depletion of distinct subunits as indicated.
(PDF)

S3 Fig The BAF250a, but not BAF250b-containing BAF complex, is specifically required for maintenance of repression of latent HIV. (A) J-Lat A2 cells latently infected with LTR-Tat-IRES GFP virus were nucleofected with either control siRNA or siRNAs targeting BAF250a, BAF250b, and BRG-1 as indicated. GFP mRNA expression was determined by RT-PCR at the times indicated after transfection, was normalized to GAPDH, and is presented as fold increase over untransfected control. (B) RT-PCR analysis indicated stable depletion of ARID1B/BAF250b mRNA up to 6 d after siRNA transfection. Error bars represent the SEM of three independent experiments. * *p* < 0.05, ** *p* < 0.01.
(PDF)

S4 Fig BAF180-facilitated Tat activation of the LTR is dependent on Tat residues K50,51. (A) CMV-driven luciferase activity is not affected by the depletion of either BAF180 or BAF250a. Jurkat cells were nucleofected with siRNAs against BAF180, BAF250, or with a control siRNA pool. Forty-eight hours after siRNA treatment, cells were transfected with a CMV-luciferase vector. (B) Transactivation of the HIV promoter by wild-type but not K50,51 mutant Tat is reduced in the absence of BAF180. Jurkat cells containing integrated LTR-Luciferase (LTR-Luc) were nucleofected with siRNAs against BAF180, BAF250, or with a control siRNA pool. After 48 h, cells were re-transfected with either a control, a CMV-driven wild-type Tat or K50,51R mutant Tat-expression vector. Luciferase was measured after 24 h. Error bars represent the SEM of three independent experiments. * *p* < 0.05.
(PDF)

S5 Fig Analysis of chromatin structure and direct binding of distinct SWI/SNF complexes to the HIV promoter before and after PMA stimulation in J-Lat A2. (A) PMA stimulation causes increase in DNA accessibility over the LTR nuc-1. FAIRE results are presented as fold change respective to unstimulated value for each primer pair. (B) PMA stimulation is accompanied by reduction in histone density over LTR nuc-1 as determined by H3 and H2B ChIPs. Histone ChIP results are presented as fold change (histone/mock IP) respective to unstimulated value for each primer pair. (C) BAF directly binds to nuc-1 in its repressed state, while PBAF is recruited to nuc-1 upon PMA stimulation. SWI/SNF subunit ChIPs are presented as ratio of immunoprecipitated DNA over input. Immunoprecipitated DNA from ChIPs and phenol:chloroform extracted DNA from FAIRE were analyzed by qPCR using primer pairs specific for nuc-0, DHS1, and nuc-1 LTR regions. For all ChIP and FAIRE experiments, error bars represent the SEM of at least three independent experiments. * *p* < 0.05, ** *p* < 0.01.
(PDF)

S6 Fig BAF positions nuc-1 of HIV LTR. (A) Location of strictly positioned nucleosomes correlate negatively with the predicted histone binding affinity score (nucleosome score) of the DNA sequence encompassing the HIV LTR. Similarity of the predicted nucleosome affinity for HIV nucleotide sequence 1–1800 determined using the algorithm described in (Xi et al., 2010 [55]) (shown in black) and an alternative algorithm described in (Kaplan et al., 2009 [56]; Segal et al., 2006 [57]) (shown in red). (B) Depletion of BAF250 and BRG1 results in a peak in DNA accessibility over LTR nuc-1. J-Lat A2 cells were nucleofected with either control nontargeting siRNA or siRNAs targeting individual SWI/SNF subunits as indicated and subjected to FAIRE after 72 h. FAIRE results are presented as fold change respective to the value obtained for control siRNA transfected cells given a value of 1 for each primer pair. (C) Depletion of BAF results in reduced histone density over nuc-1 of HIV LTR as determined by H3 and H2B ChIPs. Histone ChIP results are presented as percent immunoprecipitated over Input. For all ChIP and FAIRE experiments error bars represent the SEM of at least three independent experiments. * *p* < 0.05, ** *p* < 0.01.
(PDF)

S7 Fig BAF promotes the establishment of latent HIV infections in SupT1 cells. (A) Top panel: schematic representation of protocol for comparison of productive and latent infections in SupT1 cells containing or depleted of remodeling complex subunits. SupT1 cells were first nucleofected with control siRNA or siRNA targeting individual SWI/SNF subunits as indicated. After 48 h, cells were infected with retroviral particles containing the vector LTR-Tat-IRES-GFP. The percentage of productive infections (GFP-positive cells obtained after infection (16.2%) (top panel)), FACS-sorted GFP-negative cells (middle panel), or percent latent infections (GFP-positive cells obtained after PMA treatment of sorted GFP-negative cells (2.63%) (bottom panel)) are shown for SupT1 cells nucleofected with control siRNA. (B) Depletion of BAF/PBAF subunits as indicated does not significantly affect the percentage of productive HIV infections. (C) Depletion of the core SWI/SNF subunits BRG1 and INI-1 and the BAF-specific subunit BAF250 significantly decreases the incidence of latent HIV infections (the percent GFP positive cells obtained after PMA stimulation of GFP negative cell population) while depletion of PBAF-specific subunits BAF200 or BAF180 had no significant effect on latency establishment. (D) Western blot analysis demonstrates depletion of BAF/PBAF subunits as indicated 96 h after siRNA transfection.
(PDF)

S8 Fig BAF and CHD3 do not synergize in promoting the establishment of latent HIV infections. Jurkat cells (A-C) or SupT1 cells (D-F) were first nucleofected with control siRNA or siRNA targeting BAF250, BRG1, CHD3, BAF250 together with CHD3, or BRG1 together with CHD3. After 48 hours, cells were infected with retroviral particles containing the vector LTR-Tat-IRES-GFP. The percentages of productive or latent infections were determined as described in Fig 8 and S7 Fig. Depletion of CHD3 and BAF subunits alone or together with CHD3 does not significantly affect the percentage of productive HIV infections in either Jurkat (A) or SupT1 (D) cells. Depletion of BAF subunits BRG1 and BAF250 and the Mi2 catalytic subunit CHD3 significantly decreases the incidence of latent HIV infections. However, simultaneous depletion of BAF and CHD3 does not result in an additive decrease in latency establishment in Jurkat (B) or SupT1 (E) cells. Western blotting analysis indicates depletion of the indicated remodeling subunits in Jurkats ((C) and Fig 8A) and SupT1 ((F) and S7D Fig) cells 96 h post-siRNA transfection.
(PDF)

S1 Table Primer pairs used to analyze ChIP, FAIRE, and MNase experiments by qPCR. (PDF)
