# Correction: Repressive LTR Nucleosome Positioning by the BAF Complex Is Required for HIV Latency

**DOI:** 10.1371/journal.pbio.1002302

**Published:** 2015-11-16

**Authors:** Haleh Rafati, Maribel Parra, Shweta Hakre, Yuri Moshkin, Eric Verdin, Tokameh Mahmoudi

## Notice of Republication

Due to the publication of copyrighted material, this article has been republished in order to remove this content from Fig 4, and from the published record. Please see below for full details.

In addition to this, the authors would like to make the following corrections and clarifications to Fig 2 and Fig 4, as well as to Supporting S1 and S8 Figs. The corrections are as follows:

1. In Fig 2, due to errors in gel loading order for the BAF-180 and BAF-250 knock-down time-course western blot analysis, the affected sample lanes were spliced into the correct order when the Fig 2D image was created. The β-actin lanes were, however, not correspondingly spliced in the originally published version as the signal was constant and did not change upon BAF-180 or BAF-250 depletion. In the corrected Fig 2D, we now clearly indicate all the relevant splice lines and have also spliced the β-actin lanes corresponding to the BAF-180 and BAF-250 panels to follow the same splicing pattern. The conclusions from this figure are not affected.
  The corrected Fig 2 file is provided.
2. In Fig 4 the two FLAG-Tat panels within panel 4A were intentionally duplicated for presentation purposes in the originally published figure. They demonstrated expression of Tat in the A2 cell line after PMA treatment. These panels were included for presentation purposes as the same starting material (-/+ Tat) is used for both the Input and α-FLAG IP lanes. Due to this confusing presentation, the corrected figure now no longer includes the FLAG-Tat panels and instead includes an α-FLAG panel from a new replicate experiment (see below).
  The originally published version of Fig 4A included FLAG-Tat, INI-1 and PKD panels that we had previously published in another journal (J Biol Chem. 2006 Jul 21;281(29):19960–8). They were also included in this PLOS Biology publication as controls for the specific Tat interaction with PBAF-specific BAF180 and not BAF250, which is the novelty of Fig 4 in this manuscript. When discussing this figure in article text, we had referred to the experiment and controls published in the JBC paper. However, due to reasons of copyright, we have now performed replicate experiments and in corrected Fig 4A we include this new data to replace the data that had previously been published elsewhere. The new replicate data provide the same results as the originally published version and the conclusions are unaffected.
  The corrected Fig 4 file is provided.
3. S1 Fig is being corrected to indicate a splice line between the PMA lane and the NaN3 1mM lane in panel S1B.
  The corrected S1 Fig file is provided.
4. In S8 Fig the BAF250 panel (and its control Tubulin panel) and the BRG-1 panel (and its control Tubulin panel) from Figure 8A are intentionally duplicated in S8C Fig for presentation purposes and easy comparison. Similarly, the BAF250 panel (and its control Tubulin panel) and the BRG-1 panel (and its control Tubulin panel) from Figure S7D are also intentionally duplicated in S8F Fig for presentation purposes and easy comparison. To make the figure legend text more explicit than it currently is, we have added this clarification wording to the corrected S8 Fig legend. The figures remain unchanged.
  The corrected legend for S8 Fig is provided.

**Fig 2:**
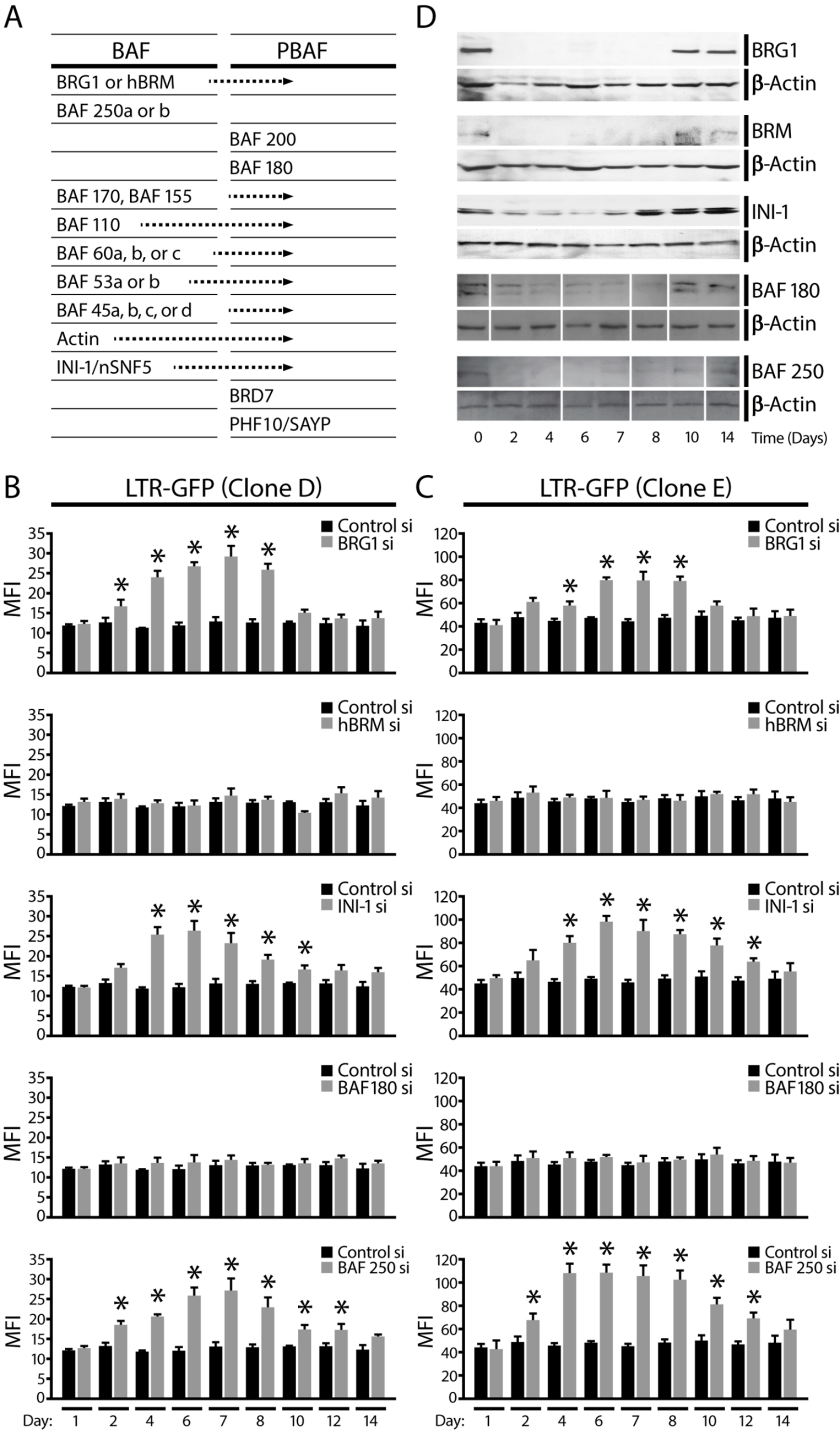
The BAF complex represses basal transcription at the HIV promoter. (A) Table of subunit composition of the two distinct SWI/SNF complexes, BAF and PBAF, in mammals. (B) GFP expression was monitored by flow cytometry at indicated times after siRNA transfection to measure HIV promoter activity. Results are presented as MFI for cells treated with a control siRNA or siRNAs specific for SWI/SNF subunits. (C) Same experiments as shown in (B) for clone D, with clone E, another Jurkat cell line containing an integrated LTR-GFP virus. Error bars represent the SEM of five independent experiments. * *p*<0.05. (D) Jurkat cells containing an integrated LTR-GFP virus (clone D) were transfected with control siRNA or siRNAs targeting various SWI/SNF complex subunits as indicated. Western blot analysis shows expression of each SWI/SNF subunit after its specific depletion 0, 2, 4, 6, 7, 8, 10, and 14 d after siRNA transfection with each specific antibody and β-actin loading control as indicated.

**Fig 4:**
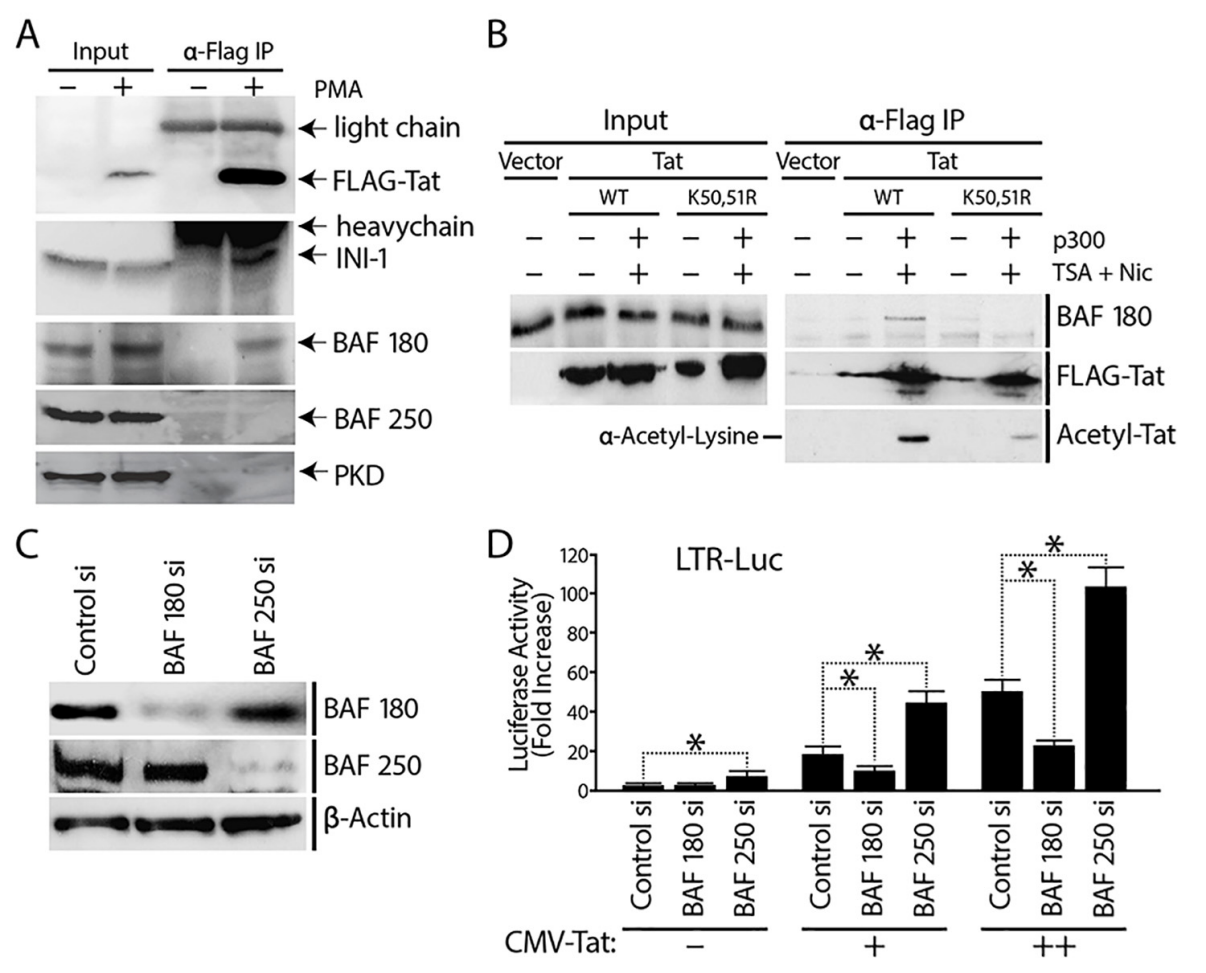
PBAF, recruited by K50K51 acetylated Tat, is a co-factor for Tat activation of the HIV promoter. (A) J-Lat A2 cells containing an integrated LTR-Tat-FLAG-GFP were stimulated with PMA to induce expression of Tat-FLAG. Tat was immunoprecipitated from untreated or PMA-stimulated cell lysates and its associated proteins were examined by SDS-PAGE and Western blotting with antibodies against the BAF- or PBAF-specific subunits BAF250a and BAF180, and protein kinase D-1 and 14-3-3 as controls. (B) Tat co-immunoprecipitation with BAF180 is modulated by Tat acetylation. Tat (wild-type or K50R/L51R) was immunoprecipitated using anti-FLAG antibody and analyzed by Western blotting using antibody specific for BAF180. Tat acetylation levels were assessed using an anti-acetyl lysine antibody. All proteins were expressed at similar levels under the different experimental conditions as shown by the Inputs. (C) 1G5 Jurkat cells containing integrated LTR-Luciferase (LTR-Luc) were nucleofected with siRNAs against BAF180, BAF250, or with a control siRNA pool. Expression of BAF180, BAF250, and β-actin was analyzed by Western blotting after depletion of either BAF180 or BAF250. (D) Transactivation of the HIV promoter by Tat is reduced in the absence of BAF180. 48 h after siRNA depletion of BAF180 or BAF250, cells were re-transfected with either a control or Tat-expression vector (CMV-driven), and luciferase assay performed after 24 h. Error bars represent the SEM of three independent experiments. * *p*<0.05. (D).

## Supporting Information

S1 Fig ATP depletion results in nuc-1 remodeling and HIV promoter activation. (A) Schematic representation of the restriction sites and probe used to analyze the remodeling of nuc-1. Nuclei isolated from cells treated either with PMA or sodium azide (NaN_3_) were digested in vitro with AflII to probe for accessibility of the DNA encompassing nuc-1. Genomic DNA was subsequently digested with *Nco*I in vitro, and the DNA was analyzed by indirect-end labeling. The *Nco*I genomic fragment (fragment B) and the double *Nco*I */AflII* digestion product (fragment A) are shown. (B) Indirect-end labeling after PMA or NaN_3_ treatment and (C) corresponding increase in GFP expression in Jurkat clone D containing an integrated LTR-GFP virus. (D) Indirect-end labeling after NaN_3_ treatment and (E) corresponding increase in GFP expression in J-Lat A2 containing an integrated latent LTR-Tat-IRES-GFP virus. GFP, measured by flow cytometry, is shown as mean fluorescence intensity (MFI) (C) or increase in percent GFP positive cells (E) 16 h after treatment as detailed above. The intensities of bands from three experiments were quantitated using Odyssey software and used to compare fold increase in ratio of bands A/B in each condition and plotted as mean ± SEM. * *p*<0.05, ** *p*<0.01.
(TIF)

S8 Fig BAF and CHD3 do not synergize in promoting the establishment of latent HIV infections. Jurkat cells (A-C) or SupT1 cells (D-F) were first nucleofected with control siRNA or siRNA targeting BAF250, BRG1, CHD3, BAF250 together with CHD3, or BRG1 together with CHD3. After 48 hours, cells were infected with retroviral particles containing the vector LTR-Tat-IRES-GFP. The percentages of productive or latent infections were determined as described in Figures 8 and S7. Depletion of CHD3 and BAF subunits alone or together with CHD3 does not significantly affect the percentage of productive HIV infections in either Jurkat (A) or SupT1 (D) cells. Depletion of BAF subunits BRG1 and BAF250 and the Mi2 catalytic subunit CHD3 significantly decreases the incidence of latent HIV infections. However, simultaneous depletion of BAF and CHD3 does not result in an additive decrease in latency establishment in Jurkat (B) or SupT1 (E) cells. Western blotting analysis indicates depletion of the indicated remodeling subunits in Jurkats ((C) and Fig 8A) and SupT1 ((F) and Fig S7D) cells 96 h post-siRNA transfection.
(PDF)
