# Supplementary figures and images for: Repressive LTR Nucleosome Positioning by the BAF Complex Is Required for HIV Latency
Source: PLoS Biol. 2011 Nov 29;9(11):e1001206. doi: 10.1371/journal.pbio.1001206 (PMC3226458; doi:10.1371/journal.pbio.1001206)

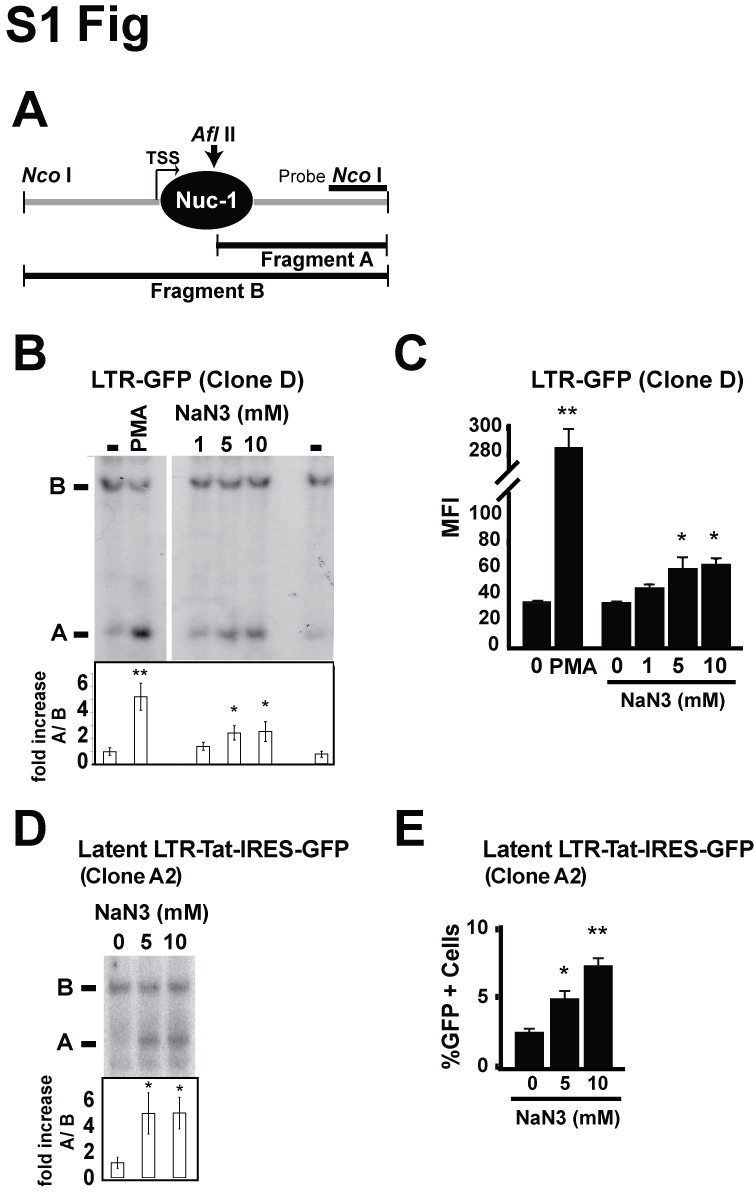

Supplement: S1 Fig — (A) Schematic representation of the restriction sites and probe used to analyze the remodeling of nuc-1. Nuclei isolated from cells treated either with PMA or sodium azide (NaN3) were digested in vitro with AflII to probe for accessibility of the DNA encompassing nuc-1. Genomic DNA was subsequently digested with NcoI in vitro, and the DNA was analyzed by indirect-end labeling. The NcoI genomic fragment (fragment B) and the double NcoI/AflII digestion product (fragment A) are shown. (B) Indirect-end labeling after PMA or NaN3 treatment and (C) corresponding increase in GFP expression in Jurkat clone D containing an integrated LTR-GFP virus. (D) Indirect-end labeling after NaN3 treatment and (E) corresponding increase in GFP expression in J-Lat A2 containing an integrated latent LTR-Tat-IRES-GFP virus. GFP, measured by flow cytometry, is shown as mean fluorescence intensity (MFI) (C) or increase in percent GFP positive cells (E) 16 h after treatment as detailed above. The intensities of bands from three experiments were quantitated using Odyssey software and used to compare fold increase in ratio of bands A/B in each condition and plotted as mean ± SEM. * p<0.05, ** p<0.01. (TIF) [file pbio.1001206.s001.tif]

## Figure S2

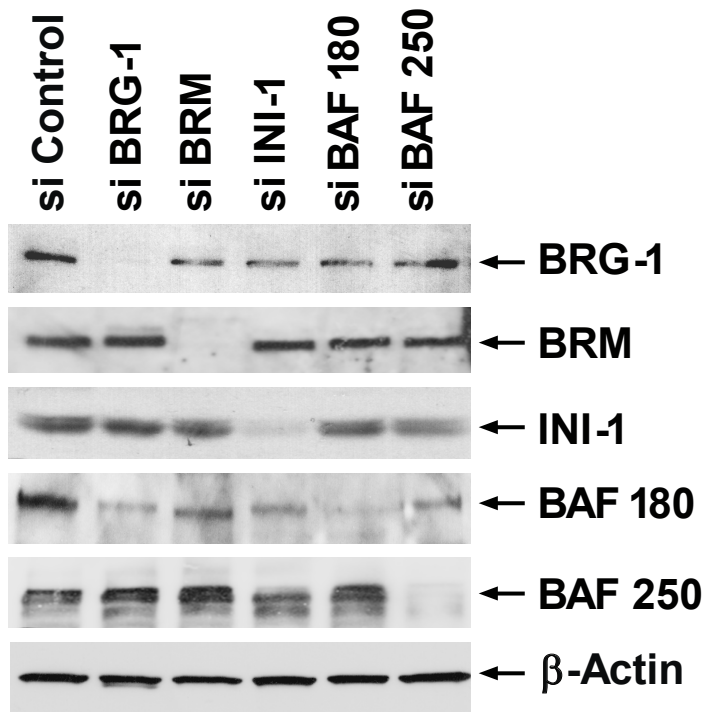

Supplement: S2 Fig — Jurkat cells containing an integrated LTR-GFP virus (clone D) were transfected with either control siRNA or siRNAs targeting various SWI/SNF complex subunits as indicated. Western blot analysis shows expression of SWI/SNF complex subunits in presence of siRNA depletion of distinct subunits as indicated. (PDF) [file pbio.1001206.s002.pdf]

# Figure S3

## A

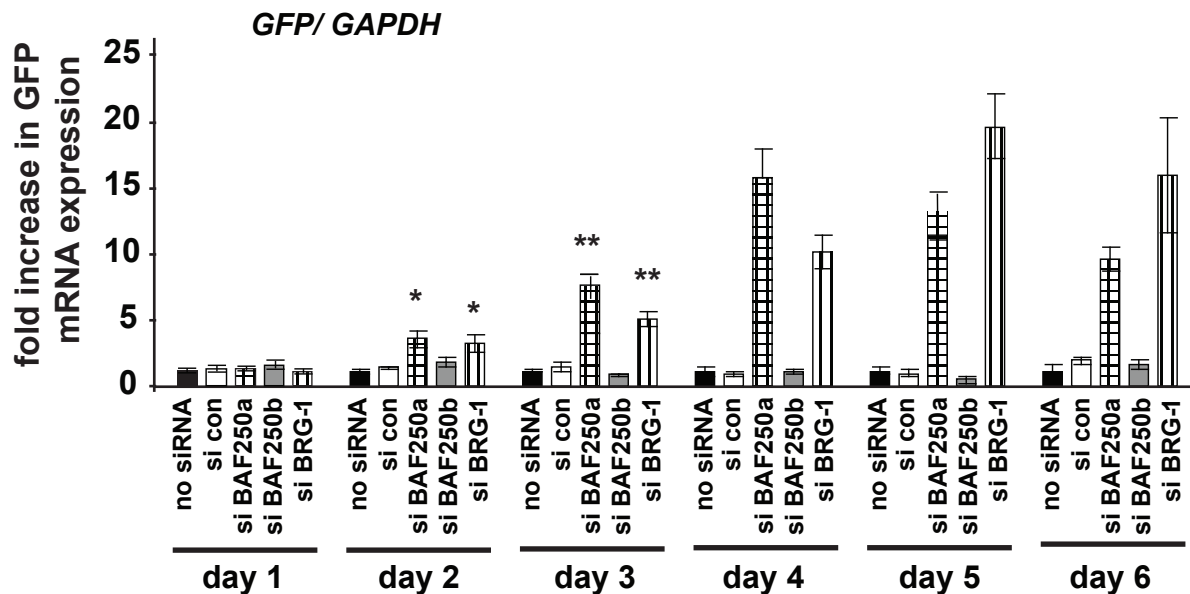

## B

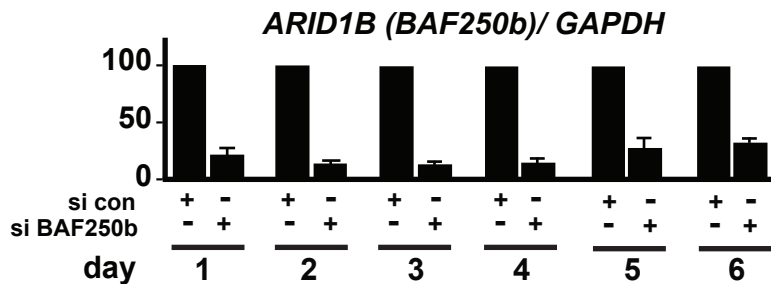

Supplement: S3 Fig — (A) J-Lat A2 cells latently infected with LTR-Tat-IRES GFP virus were nucleofected with either control siRNA or siRNAs targeting BAF250a, BAF250b, and BRG-1 as indicated. GFP mRNA expression was determined by RT-PCR at the times indicated after transfection, was normalized to GAPDH, and is presented as fold increase over untransfected control. (B) RT-PCR analysis indicated stable depletion of ARID1B/BAF250b mRNA up to 6 d after siRNA transfection. Error bars represent the SEM of three independent experiments. * p < 0.05, ** p < 0.01. (PDF) [file pbio.1001206.s003.pdf]

# Figure S4

## A

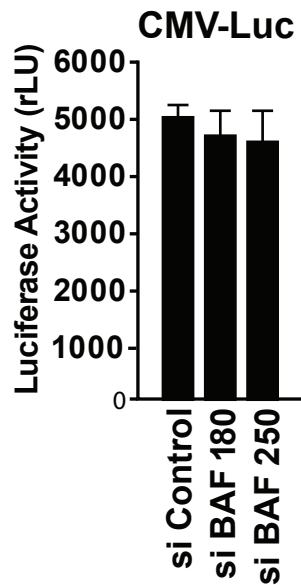

## B

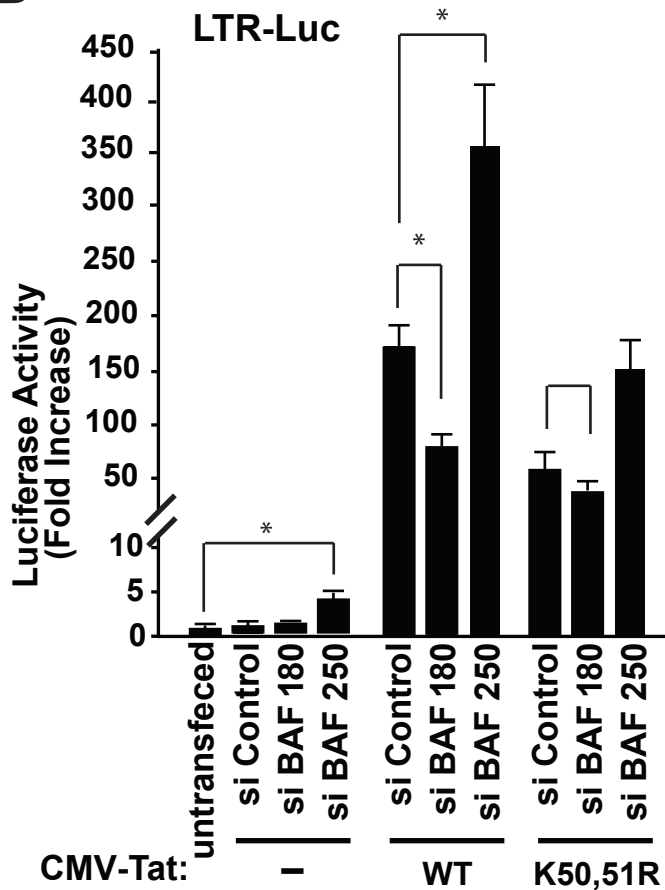

Supplement: S4 Fig — (A) CMV-driven luciferase activity is not affected by the depletion of either BAF180 or BAF250a. Jurkat cells were nucleofected with siRNAs against BAF180, BAF250, or with a control siRNA pool. Forty-eight hours after siRNA treatment, cells were transfected with a CMV-luciferase vector. (B) Transactivation of the HIV promoter by wild-type but not K50,51 mutant Tat is reduced in the absence of BAF180. Jurkat cells containing integrated LTR-Luciferase (LTR-Luc) were nucleofected with siRNAs against BAF180, BAF250, or with a control siRNA pool. After 48 h, cells were re-transfected with either a control, a CMV-driven wild-type Tat or K50,51R mutant Tat-expression vector. Luciferase was measured after 24 h. Error bars represent the SEM of three independent experiments. * p < 0.05. (PDF) [file pbio.1001206.s004.pdf]

# Figure S5

**A**

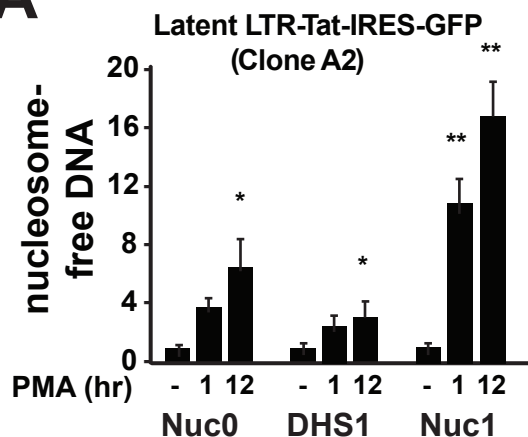

**C**

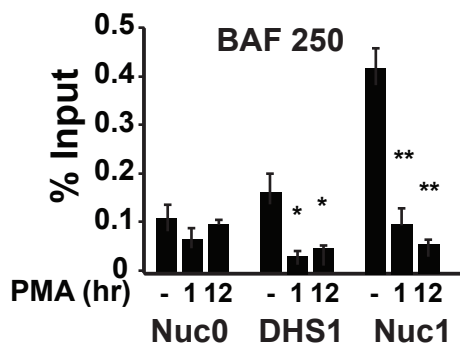

**B**

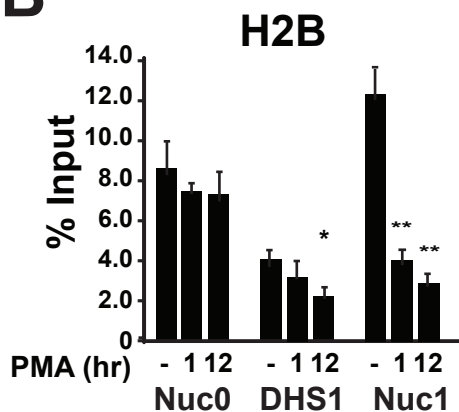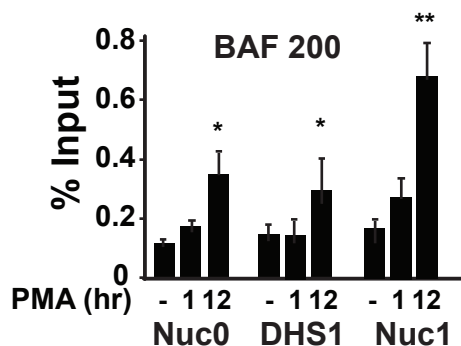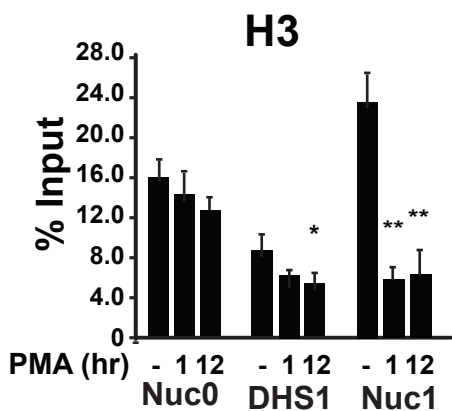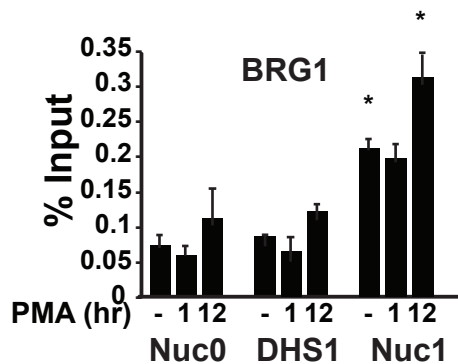

Supplement: S5 Fig — (A) PMA stimulation causes increase in DNA accessibility over the LTR nuc-1. FAIRE results are presented as fold change respective to unstimulated value for each primer pair. (B) PMA stimulation is accompanied by reduction in histone density over LTR nuc-1 as determined by H3 and H2B ChIPs. Histone ChIP results are presented as fold change (histone/mock IP) respective to unstimulated value for each primer pair. (C) BAF directly binds to nuc-1 in its repressed state, while PBAF is recruited to nuc-1 upon PMA stimulation. SWI/SNF subunit ChIPs are presented as ratio of immunoprecipitated DNA over input. Immunoprecipitated DNA from ChIPs and phenol:chloroform extracted DNA from FAIRE were analyzed by qPCR using primer pairs specific for nuc-0, DHS1, and nuc-1 LTR regions. For all ChIP and FAIRE experiments, error bars represent the SEM of at least three independent experiments. * p < 0.05, ** p < 0.01. (PDF) [file pbio.1001206.s005.pdf]

Figure S6

A

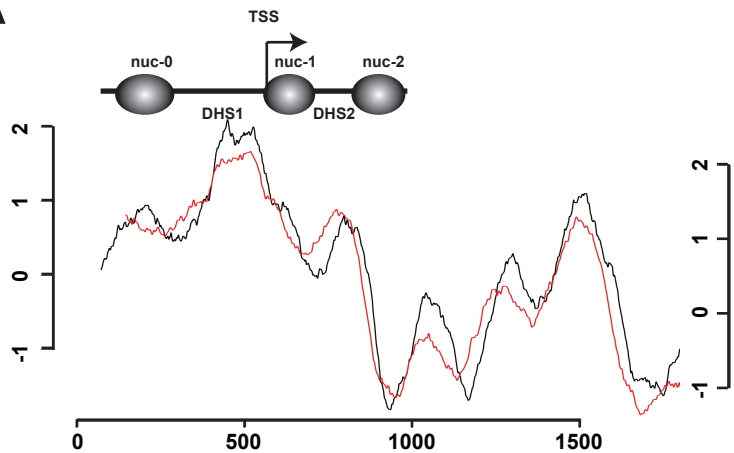

B

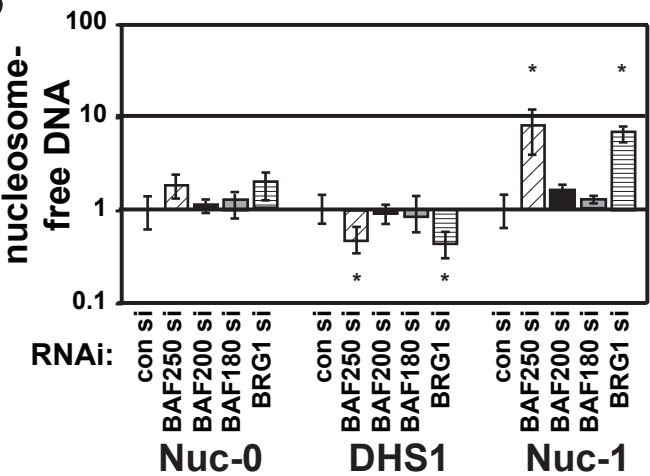

C

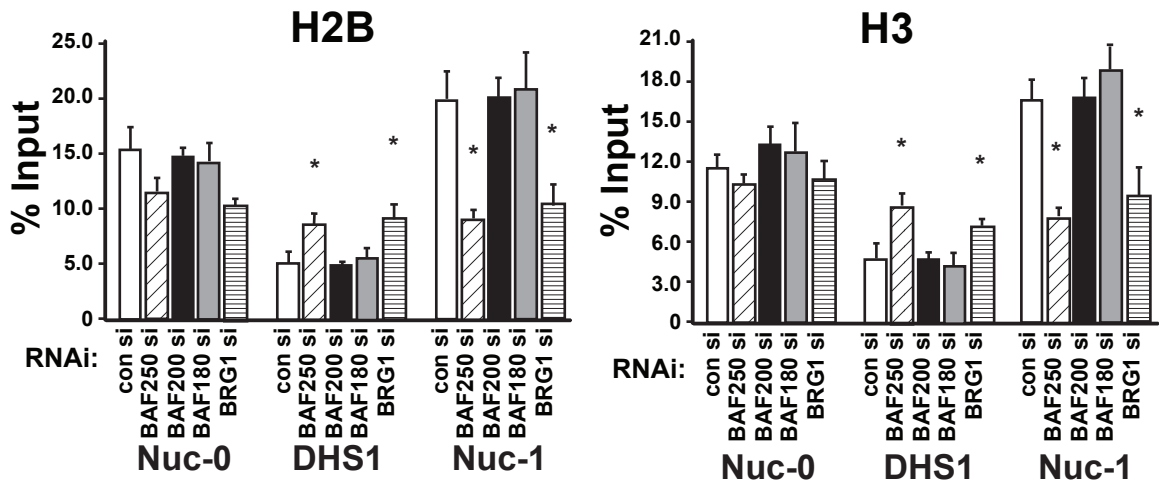

Supplement: S6 Fig — (A) Location of strictly positioned nucleosomes correlate negatively with the predicted histone binding affinity score (nucleosome score) of the DNA sequence encompassing the HIV LTR. Similarity of the predicted nucleosome affinity for HIV nucleotide sequence 1–1800 determined using the algorithm described in (Xi et al., 2010 [55]) (shown in black) and an alternative algorithm described in (Kaplan et al., 2009 [56]; Segal et al., 2006 [57]) (shown in red). (B) Depletion of BAF250 and BRG1 results in a peak in DNA accessibility over LTR nuc-1. J-Lat A2 cells were nucleofected with either control nontargeting siRNA or siRNAs targeting individual SWI/SNF subunits as indicated and subjected to FAIRE after 72 h. FAIRE results are presented as fold change respective to the value obtained for control siRNA transfected cells given a value of 1 for each primer pair. (C) Depletion of BAF results in reduced histone density over nuc-1 of HIV LTR as determined by H3 and H2B ChIPs. Histone ChIP results are presented as percent immunoprecipitated over Input. For all ChIP and FAIRE experiments error bars represent the SEM of at least three independent experiments. * p < 0.05, ** p < 0.01. (PDF) [file pbio.1001206.s006.pdf]

# Figure S7

**A**

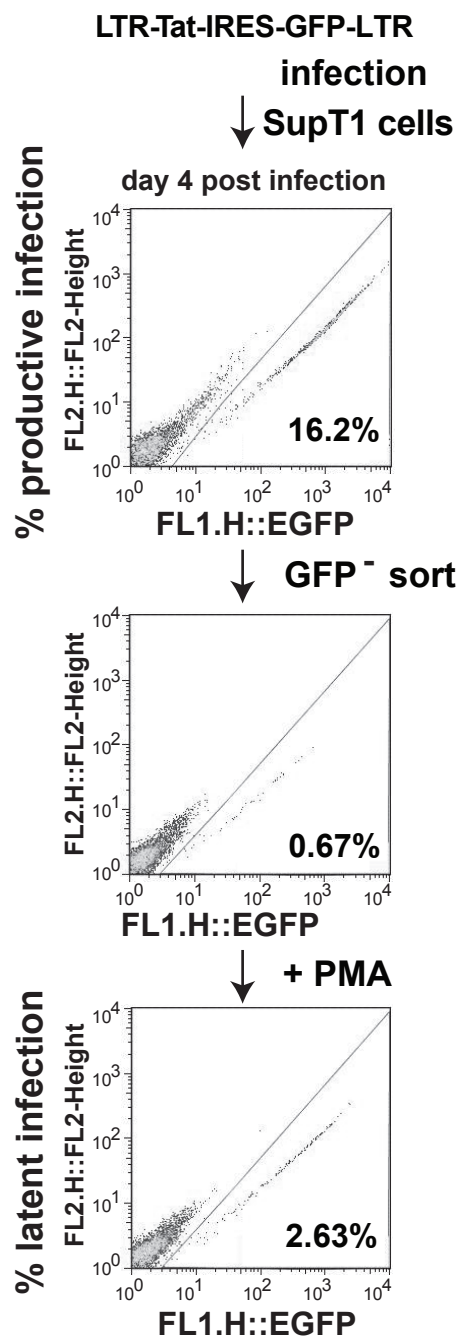

**B**

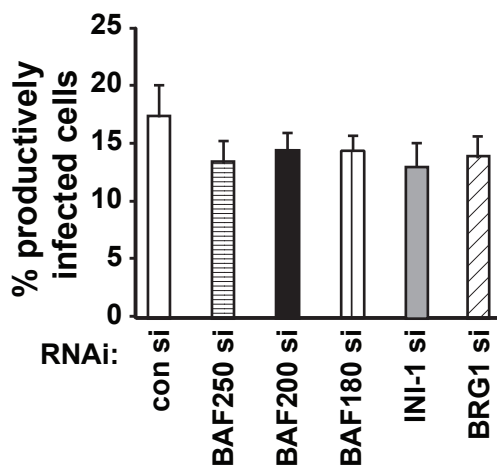

**C**

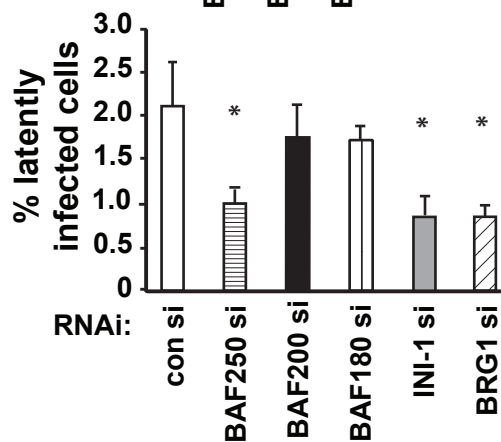

**D**

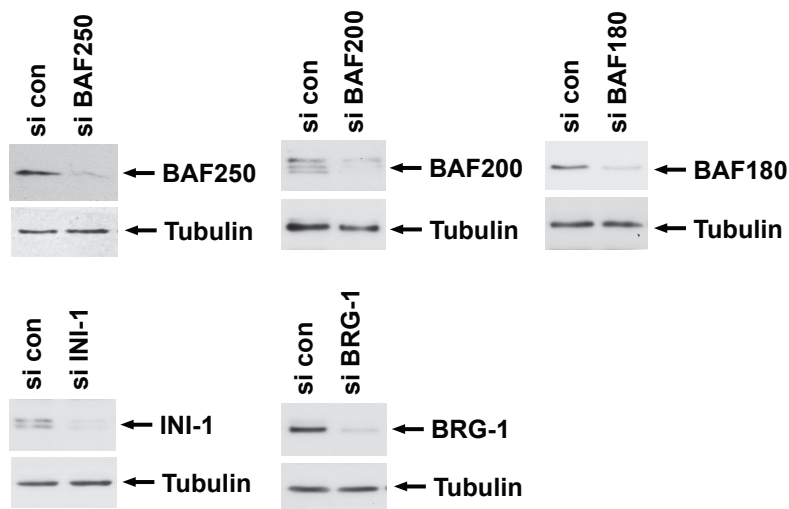

Supplement: S7 Fig — (A) Top panel: schematic representation of protocol for comparison of productive and latent infections in SupT1 cells containing or depleted of remodeling complex subunits. SupT1 cells were first nucleofected with control siRNA or siRNA targeting individual SWI/SNF subunits as indicated. After 48 h, cells were infected with retroviral particles containing the vector LTR-Tat-IRES-GFP. The percentage of productive infections (GFP-positive cells obtained after infection (16.2%) (top panel)), FACS-sorted GFP-negative cells (middle panel), or percent latent infections (GFP-positive cells obtained after PMA treatment of sorted GFP-negative cells (2.63%) (bottom panel)) are shown for SupT1 cells nucleofected with control siRNA. (B) Depletion of BAF/PBAF subunits as indicated does not significantly affect the percentage of productive HIV infections. (C) Depletion of the core SWI/SNF subunits BRG1 and INI-1 and the BAF-specific subunit BAF250 significantly decreases the incidence of latent HIV infections (the percent GFP positive cells obtained after PMA stimulation of GFP negative cell population) while depletion of PBAF-specific subunits BAF200 or BAF180 had no significant effect on latency establishment. (D) Western blot analysis demonstrates depletion of BAF/PBAF subunits as indicated 96 h after siRNA transfection. (PDF) [file pbio.1001206.s007.pdf]

# Figure S8

**A**

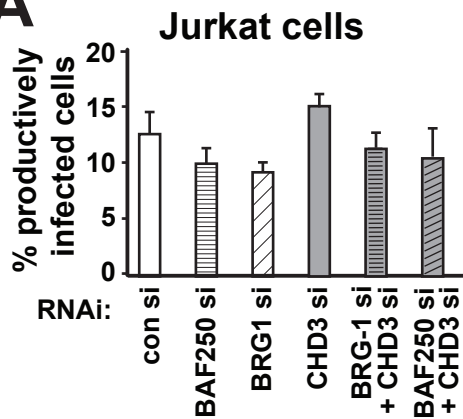

**B**

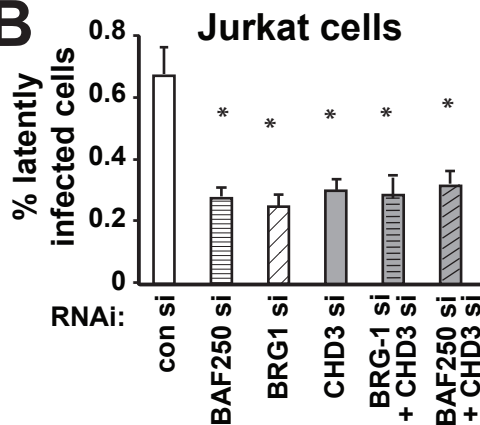

**C**

**Jurkat cells**

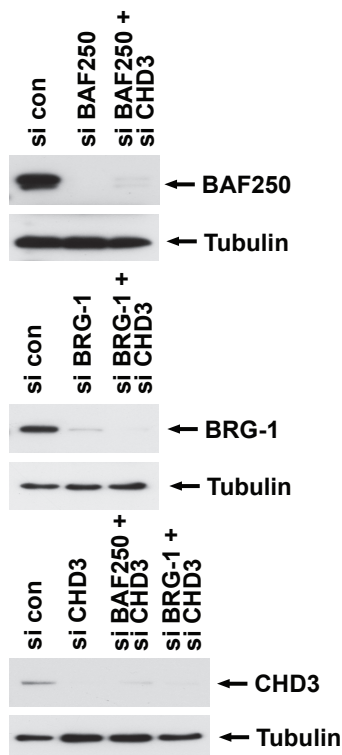

**D**

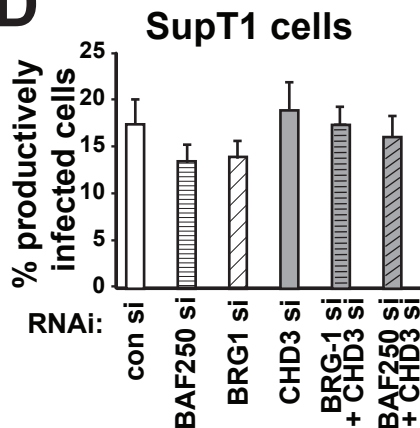

**E**

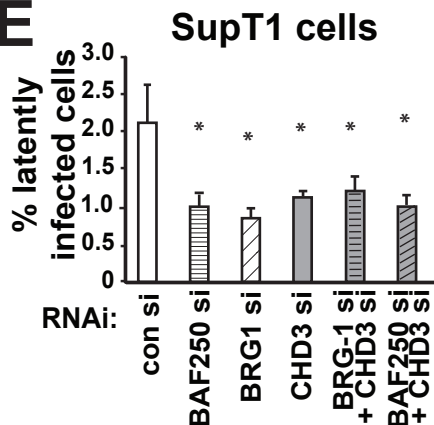

**F**

**SupT1 cells**

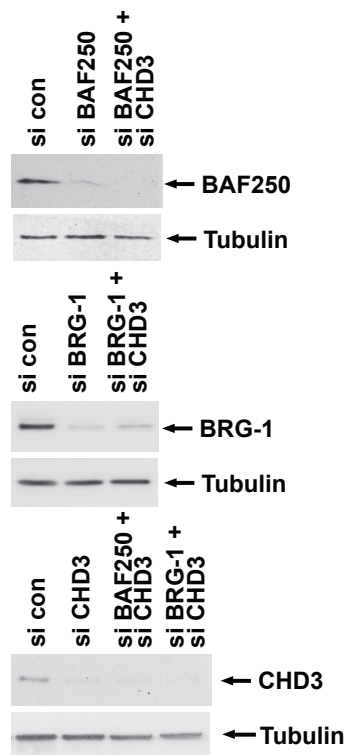

Supplement: S8 Fig — Jurkat cells (A-C) or SupT1 cells (D-F) were first nucleofected with control siRNA or siRNA targeting BAF250, BRG1, CHD3, BAF250 together with CHD3, or BRG1 together with CHD3. After 48 hours, cells were infected with retroviral particles containing the vector LTR-Tat-IRES-GFP. The percentages of productive or latent infections were determined as described in Fig 8 and S7 Fig. Depletion of CHD3 and BAF subunits alone or together with CHD3 does not significantly affect the percentage of productive HIV infections in either Jurkat (A) or SupT1 (D) cells. Depletion of BAF subunits BRG1 and BAF250 and the Mi2 catalytic subunit CHD3 significantly decreases the incidence of latent HIV infections. However, simultaneous depletion of BAF and CHD3 does not result in an additive decrease in latency establishment in Jurkat (B) or SupT1 (E) cells. Western blotting analysis indicates depletion of the indicated remodeling subunits in Jurkats ((C) and Fig 8A) and SupT1 ((F) and S7D Fig) cells 96 h post-siRNA transfection. (PDF) [file pbio.1001206.s008.pdf]
