# Supplementary material for: Repressive LTR Nucleosome Positioning by the BAF Complex Is Required for HIV Latency
Source: PLoS Biol. 2011 Nov 29;9(11):e1001206. doi: 10.1371/journal.pbio.1001206 (PMC3226458; doi:10.1371/journal.pbio.1001206)
Supplement: S1 Table — (PDF) [file pbio.1001206.s009.pdf]

Table S1.

Primer pairs used to analyze ChIP, FAIRE, and MNase experiments by qPCR:
Nuc0F1-5'ATCTACCACACACAAGGCTAC3'
Nuc0R1-5'GTACTAACTTGAAGCACCATCC3'
Nuc0F2-5'TGGATCTACCACACACAAGG3'
Nuc0R2-5'GTACTAACTTGAAGCACCATCC3'
HSF1-5'TTACACCCTATGAGCCAGCATG2'
HSR1-5'GCTCTCGGGCCATGTGAC3'
HSF2-5'AAGTTTGACAGCCTCCTAGC3'
HSR2-5'CACACCTCCCTGGAAAGTC3'
Nuc1F1-5'TCTCTGGCTAACTAGGGAACC3'
Nuc1R1-5'AAAGGGTCTGAGGGATCTCTAG3'
Nuc1F2-5'TCTCTGGCTAACTAGGGAACC3'
Nuc1R2-5'CTAAAAGGGTCTGAGGGATCTC3'
Nuc1F3-5'GAGCCTGGGAGCTCTCTG3'
Nuc1R3-5'GCTAGAGATTTTCCACACTG3'
Nuc2-F1-5'GCGGAGGCTAGAAGGAGAGAG3'
Nuc2-R1-5'GCTCCCTGCTTGCCCATAC3'
Nuc2-F2-5'AGAGATGGGTGCGAGAGC3'
Nuc2-R2-5'ATTAACTGCGAATCGTTCTAGC3'
Control primer pairs used in ChIP – control upstream region of *AXIN2* gene:
Axin2 F-1-5'GCCAGAGTCAAGCCAGTAGTC3'
Axin2 R-1-5'TAGCCTAATGTGGAGTGGATGTG3'
Axin2 F-2-5'GAAGGTGTGGAGCAATGG3'
Axin2 R-2-5'CTTGGAGGCAGGAGGTAG3'
MNase F-1-5'GATCtGTGGATCTACCACAC3'
MNase R-1-5'GCACCATCCAAAGGTCAGTGG3'
MNase F-2-5'CCTGATTGGCAGAACTACACAC3'
MNase R-2-5'TCTACTTGCTCTGGTTCAACTGG3'
MNase F-3-5'CCTTTGGATGGTGCTTCAAGTTAG3'
MNase R-3-5'ATGCTGGCTCATAGGGTGTAAC3'
MNase F-4-5'TAAGGAAGAGAAAGAACAGGCTTG3'
MNase R-4-5'GAAATGCTAGGAGGCTGTCA3'
MNase F-5-5'GAGCCAGCATGGGATGG3'
MNase R-5-5'CTCCGGATGCAGCTCTC3'
MNase F-6-5'TGACAGCCTCCTAGCATTTC3'
MNase R-6-5'CACACCTCCCTGGAAAGTC3'
MNase F-7-5'CACATGGCCCGAGAGCTG3'
MNase R-7-5'CCCAGGCCACACCTCCCTGG3'
MNase F-8-5'TACTACAAAGACTGCTGACATCG3'
MNase R-8-5'TCTGAGGGCTCGCCACTC3'
MNase F-9-5'GGGACTTTCCGCTGGGGAC3'
MNase R-9-5'CCCAGTACAGGCAAAAAGCAGC3'
MNase F-10-5'GGTGTGGCCTGGGCGGGA3'
MNase R-10-5'GTTCCCTAGTTAGCCAGAGAGC3'
MNase F-11-5'AGTGGCGAGCCCTCAGATG3'
MNase R-11-5'AGCAGTGGGTTCCCTAGTTAGC3'
MNase F-12-5'TTTGCCTGTACTGGGTCTCTCTGG3'
MNase R-12-5'CACAACAGACGGGCACACACT3'

MNase F-13-5’GCTCTCTGGCTAACTAGGGAAC3’
MNase R-13-5’AGACGGGCACACACTACTTTG3’
MNase F-14-5’AGCTCTCTGGCTAACTAGGG3’
MNase R-14-5’AAAGGGTCTGAGGGATCTCTAG3’
MNase F-15-5’TCTCTGGCTAACTAGGGAACC3’
MNase R-15-5’AAAGGGTCTGAGGGATCTCTAG3’
MNase F-16-5’AGTGTGTGCCCGTCTGTTGTG3’
MNase R-16-5’CTTTCGCTTTCAAGTCCCTGTTCG3’
MNase F-17-5’GGTAACTAGAGATCCCTCAGAC3’
MNase R-17-5’CTTCAGCAAGCCGAGTCC3’
MNase F-18-5’GTGTGGAAAATCTCTAGCAGTG3’
MNase R-18-5’CTTCAGCAAGCCGAGTCC3’
MNase F-19-5’GCGGAGGCTAGAAGGAGAGAG3’
MNase R-19-5’GCTCCCTGCTTGCCCATAC3’
MNase F-20-5’AGAGATGGGTGCGAGAGC3’
MNase R-20-5’ATTAACTGCGAATCGTTCTAGC3’

Primer pairs to generate 270 bp PCR fragment in pRRL GFP vector used in Southern Blot analysis:
EV984 5′GCCCGTCTGTTGTGTGACTCTG3′
EV987 5′CTAATTCTCCCCCGCTTAATAC3′

Primer pairs used to analyze GFP expression:
GFP F-1-5’ACGGCAAGCTGACCCTGAAG3’
GFP R-1-5’GGGTGCTCAGGTAGTGGTTG3’

Primer pairs used to analyze INO80 and 250b knock down:
INO80 F-1-5’GGACTCAGCCATGCCAAGCCCTT3’
INO80 R-1-5’GGGCTAGGCTGCTGCTCTCGTC3’
BAF250b F-5’ACCACCACCACGCACTAC3’
BAF250b R-5’TGTTGTTGGAAATGGGATGTTG3’
