# Supplementary figures and images for: Correction: Repressive LTR Nucleosome Positioning by the BAF Complex Is Required for HIV Latency
Source: PLoS Biol. 2015 Nov 16;13(11):e1002302. doi: 10.1371/journal.pbio.1002302 (PMC4646685; doi:10.1371/journal.pbio.1002302)

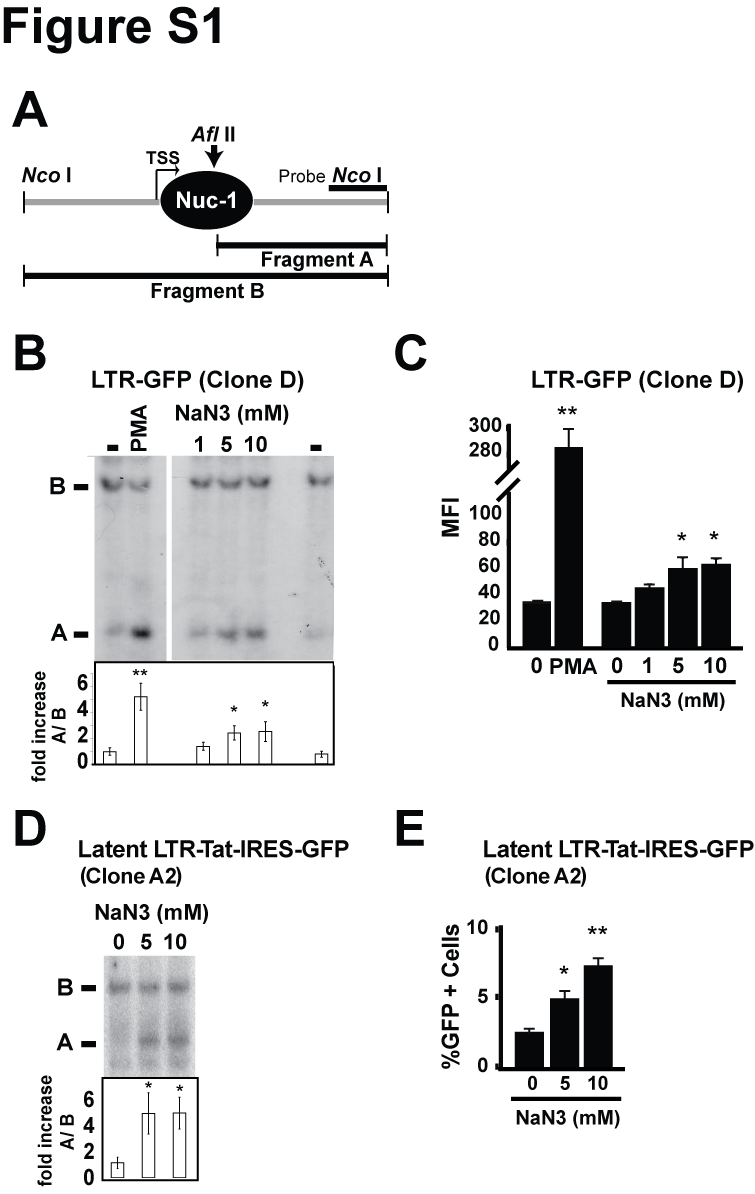

Supplement: S1 Fig — (A) Schematic representation of the restriction sites and probe used to analyze the remodeling of nuc-1. Nuclei isolated from cells treated either with PMA or sodium azide (NaN3) were digested in vitro with AflII to probe for accessibility of the DNA encompassing nuc-1. Genomic DNA was subsequently digested with NcoI in vitro, and the DNA was analyzed by indirect-end labeling. The NcoI genomic fragment (fragment B) and the double NcoI /AflII digestion product (fragment A) are shown. (B) Indirect-end labeling after PMA or NaN3 treatment and (C) corresponding increase in GFP expression in Jurkat clone D containing an integrated LTR-GFP virus. (D) Indirect-end labeling after NaN3 treatment and (E) corresponding increase in GFP expression in J-Lat A2 containing an integrated latent LTR-Tat-IRES-GFP virus. GFP, measured by flow cytometry, is shown as mean fluorescence intensity (MFI) (C) or increase in percent GFP positive cells (E) 16 h after treatment as detailed above. The intensities of bands from three experiments were quantitated using Odyssey software and used to compare fold increase in ratio of bands A/B in each condition and plotted as mean ± SEM. * p<0.05, ** p<0.01. (TIF) [file pbio.1002302.s001.tif]

# Figure S8

**A**

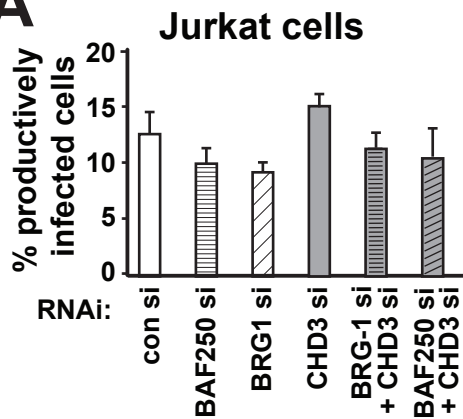

**B**

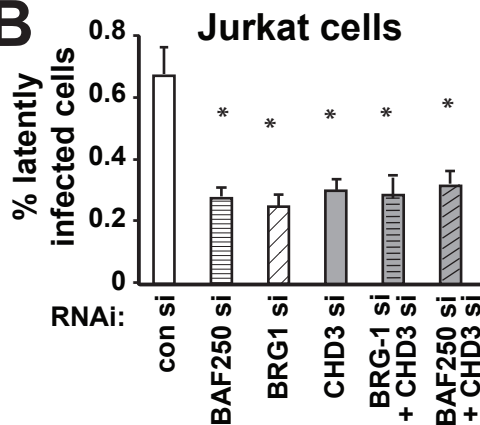

**C**

**Jurkat cells**

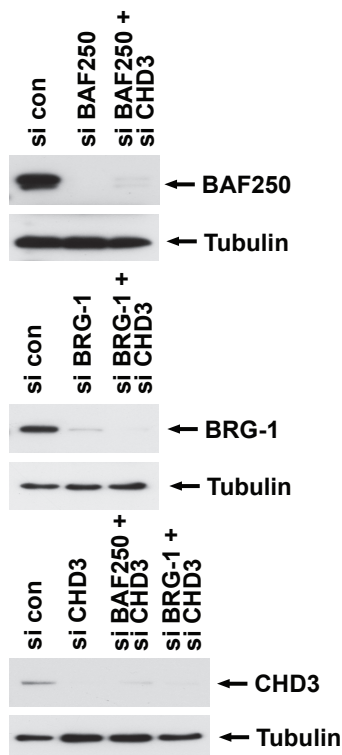

**D**

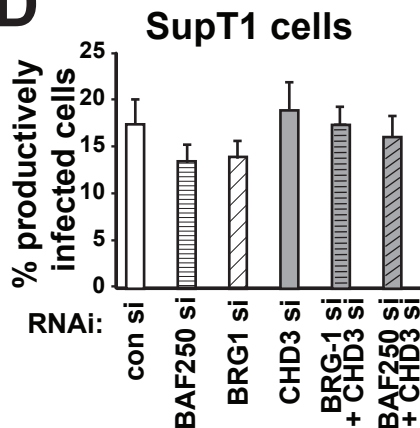

**E**

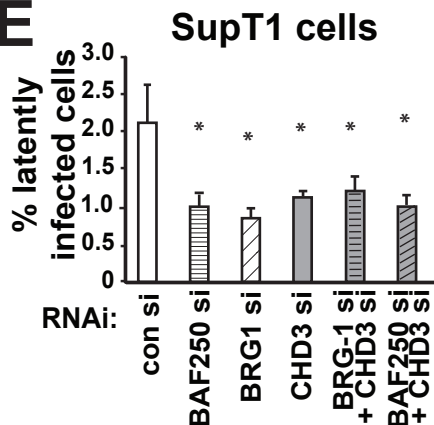

**F**

**SupT1 cells**

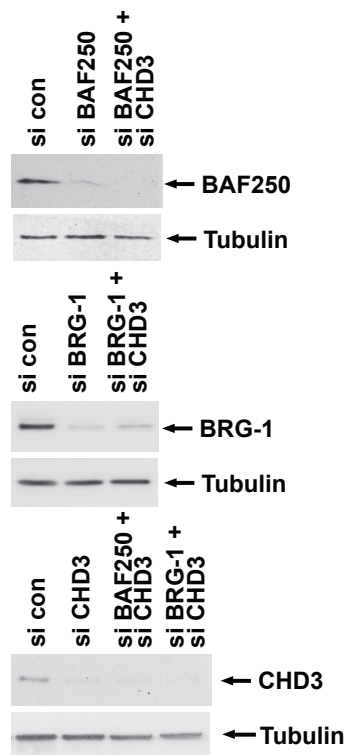

Supplement: S8 Fig — Jurkat cells (A-C) or SupT1 cells (D-F) were first nucleofected with control siRNA or siRNA targeting BAF250, BRG1, CHD3, BAF250 together with CHD3, or BRG1 together with CHD3. After 48 hours, cells were infected with retroviral particles containing the vector LTR-Tat-IRES-GFP. The percentages of productive or latent infections were determined as described in Figures 8 and S7. Depletion of CHD3 and BAF subunits alone or together with CHD3 does not significantly affect the percentage of productive HIV infections in either Jurkat (A) or SupT1 (D) cells. Depletion of BAF subunits BRG1 and BAF250 and the Mi2 catalytic subunit CHD3 significantly decreases the incidence of latent HIV infections. However, simultaneous depletion of BAF and CHD3 does not result in an additive decrease in latency establishment in Jurkat (B) or SupT1 (E) cells. Western blotting analysis indicates depletion of the indicated remodeling subunits in Jurkats ((C) and Fig 8A) and SupT1 ((F) and Fig S7D) cells 96 h post-siRNA transfection. (PDF) [file pbio.1002302.s002.pdf]
